# The neural correlates of illness awareness in addiction: a pilot exploratory analysis of preliminary data from the cognitive dysfunction in the addictions (CDiA) research program

**DOI:** 10.3389/fneur.2025.1694826

**Published:** 2026-01-05

**Authors:** Shannen Kyte, Jianmeng Song, Yuliya S. Nikolova, Anthony C. Ruocco, Sri Mahavir Agarwal, Aron Amaev, Danielle Bukovsky, Edgardo Carmona-Torres, Daniel Felsky, Ariel Graff-Guerrero, Shannon Lange, Thomas D. Prevot, Lena C. Quilty, Gary Remington, Etienne Sibille, Antonio P. Strafella, Fumihiko Ueno, Erica Vieira, Daphne Voineskos, Philip Gerretsen

**Affiliations:** 1 Institute of Medical Science, University of Toronto, Toronto, ON, Canada; 2 Multimodal Imaging Group, Research Imaging Centre, Centre for Addiction and Mental Health (CAMH), Toronto, ON, Canada; 3 Campbell Family Mental Health Research Institute, Centre for Addiction and Mental Health, Toronto, ON, Canada; 4 Department of Psychiatry, University of Toronto, Toronto, ON, Canada; 5 Department of Psychological Clinical Science, University of Toronto, Toronto, ON, Canada; 6 Department of Psychology, University of Toronto Scarborough, Toronto, ON, Canada; 7 Division of Biostatistics, Dalla Lana School of Public Health, University of Toronto, Toronto, ON, Canada; 8 Institute of Mental Health Policy Research, Centre for Addiction and Mental Health, Toronto, ON, Canada; 9 Department of Pharmacology and Toxicology, Temerty Faculty of Medicine, University of Toronto, Toronto, ON, Canada; 10 Edmond J. Safra Parkinson Disease Program, Neurology Division, Toronto Western Hospital and Krembil Brain Institute, University Health Network, Temerty Faculty of Medicine, University of Toronto, Toronto, ON, Canada

**Keywords:** impaired illness awareness, substance use disorder, alcohol use disorder, magnetic resonance imaging, functional MRI, task-based MRI, insight, anosognosia

## Abstract

**Background:**

Impaired illness awareness or anosognosia is common in substance use disorders (SUDs), including alcohol use disorder (AUD), and is a significant barrier to treatment engagement. Neuroimaging studies of the neural correlates of impaired illness awareness in other conditions have observed functional differences in regions involved in self-referential processing, namely the frontoparietal and insular areas. This study aimed to extend this research to impaired illness awareness in SUDs.

**Methods:**

Twenty participants with AUD (*n* = 10) or other SUDs (*n* = 10) (age = 35.10 (± 10.59), 80% male) were recruited from the *Cognitive Dysfunction in Addictions* (CDiA) research program at the Centre for Addiction and Mental Health in Toronto, Canada. Participants completed an illness awareness task during a functional MRI scan consisting of brief questions/statements derived from the core domains of illness awareness. Illness awareness was assessed based on response accuracy to the illness-related stimuli. Participants were grouped into impaired (≤77% response accuracy, *n* = 10) versus intact (≥77%, n = 10) illness awareness. Regression and non-parametric between-group analyses were conducted to assess brain activity, as measured by blood oxygen level dependent (BOLD) response during the illness awareness task for the AUD and other SUD groups combined and separately. Regions of interest were the posterior parietal area (PPA), dorsolateral prefrontal cortex (dlPFC), and insula.

**Results:**

Individuals with impaired illness awareness had significantly greater activation than individuals with intact illness awareness in the right insula in the AUD and SUD combined group and in the left PPA (i.e., supramarginal gyrus) in the AUD subgroup. These results were no longer significant after including illness severity as a covariate and controlling for substance category in the combined group. In the SUD subgroup, impaired illness awareness was associated with higher activation in the right dlPFC (i.e., medial superior frontal gyrus); however, this finding did not survive family-wise error correction.

**Conclusion:**

The results of this pilot exploratory study cautiously suggest that similar to other conditions that feature impaired illness awareness, subjective addiction awareness may be related to increased frontoparietal and insular brain activity during an fMRI illness awareness task. However, no findings were significant after correction for multiple testing, hence, further study in a larger sample is required to establish brain regions associated with subjective substance use awareness.

## 1 Introduction

Substance use disorder (SUD) is defined as the problematic use of a substance associated with significant negative cognitive, behavioral, social, and physiological effects (1). While effective management options are available for SUD, less than 10% of individuals sustain their engagement with treatment (2, 3). A principal barrier to treatment engagement and response is impaired illness awareness, also known as anosognosia (4, 5), or a failure to recognize the severity and negative consequences of one’s substance use (6, 7). Individuals with SUD and impaired illness awareness experience worse clinical outcomes and are less likely to engage with and continue treatment compared to those who are aware of their SUD (7–11).

Impaired illness awareness, defined by its four core domains of unawareness of one’s illness, symptoms, need for treatment, and negative consequences, has been observed in many medical and psychiatric conditions, including stroke (12, 13), Alzheimer’s disease (14, 15), schizophrenia (16–21), mood disorders (15), hypertension (22), diabetes (23), obesity (24), and addictions (25–28). Neuroimaging studies have identified structural and functional brain differences associated with impaired illness awareness (12). Seminal research, particularly in stroke patients with anosognosia, suggests this impairment may stem from an interhemispheric imbalance, specifically within frontoparietal regions (13). This theory suggests that impaired illness awareness is attributable to left hemisphere dominance stemming from either right hemisphere dysfunction or left hemisphere overactivity (13). However, more recent evidence suggests impaired illness awareness may be related to dysfunction within bilateral frontotemporoparietal and insular regions, including those in the default mode network (DMN), which support self-referential processing and introspective awareness (19, 20, 29–31). In a previous functional MRI (fMRI) study carried out by our group in patients with schizophrenia, participants were administered an illness awareness task wherein they were presented with questions/statements specific to their disorder, symptoms, and need for treatment. We found an association between impaired illness awareness and activation in the left posterior parietal area (PPA) and medial prefrontal cortex (13), suggesting that individuals with impaired illness awareness under-recruit (i.e., lesser activation) the use of frontoparietal regions at baseline, but over-recruit (i.e., greater activation) these same regions when thinking about illness-related beliefs. These results were replicated in a subsequent study where individuals with schizophrenia and impaired illness awareness had similar activation in the PPA to the same fMRI task as compared to healthy control participants (32). Moreover, another study in this same issue presents data revealing that impaired illness awareness in obesity is related to greater activity in the left PPA using the same fMRI paradigm, suggesting these brain regions may be part of a transdiagnostic subjective illness awareness network.

Few studies have investigated the neural correlates of impaired illness awareness in SUD. A recent review suggests that impaired illness awareness in SUD may involve cortical midline structures, the insula, and frontoparietal cortices (27). A key limitation of most prior imaging work is the reliance on resting-state fMRI, which cannot capture momentary neural changes occurring precisely when individuals reflect on their illness, an advantage that task-based paradigms using blood-oxygen-level-dependent (BOLD) signals offer. One study of participants with cocaine use disorder found heightened activation in the medial orbitofrontal cortex during an fMRI task designed to assess readiness for change as compared to healthy control participants (33). However, the concept of readiness for change, although related, is distinct from subjective illness awareness (33).

Given the lack of research on the neurobiological basis for impaired illness awareness in SUD, the aim of the present pilot exploratory study was to identify possible neural correlates of impaired illness awareness in SUD by assessing the core domains of illness awareness via an illness awareness task during fMRI. This study was conducted within a subset of participants within the larger *Cognitive Dysfunction in the Addictions* (CDiA) research program, which investigates cognitive dysfunction in SUD, including alcohol use disorder (AUD) as well as other SUDs (34). As such, this study uses “SUD” as an umbrella term below to refer to both AUD and other SUDs, singly and in combination. The CDiA dataset provides a unique opportunity to carry out a preliminary study to explore the neural correlates of impaired illness awareness in SUD. This study hypothesized that individuals with SUDs and impaired illness awareness will show similar areas of activation to impaired illness awareness in other conditions during an illness awareness fMRI task, particularly an increased BOLD response in frontoparietal and insular areas associated with the DMN and self-referential processing.

## 2 Methods

### 2.1 Study design

An analysis was performed of participant baseline data from the CDiA research program of a subset of individuals (*n* = 22) recruited between January 2023 to May 2024. The details of CDiA can be found in the corresponding study protocol (34). Briefly, individuals seeking treatment for substance use concerns in Toronto, Canada, were invited to participate in the observational, longitudinal CDiA research program, comprising seven interdisciplinary projects aiming to elucidate the role of cognitive impairment on functional outcomes in SUDs (34). Informed consent, written or electronic, was obtained from each participant prior to the study. The study received approval from the Research Ethics Board at CAMH.

Participants were informed to abstain from any substance, including alcohol, for at least 12 h prior to all study visits. Participants were administered a breathalyzer test immediately before any cognitive testing, including the MRI visit, to confirm zero blood alcohol concentration. Participants were rescheduled if there was an indication of substance use within that time frame.

### 2.2 Participants

The inclusion criteria for the parent CDiA research program were: (1) age 18 to 60, (2) meeting current diagnostic criteria for AUD and/or SUD (excluding nicotine- or caffeine-related disorders) based on the Diagnostic Assessment Research Tool (35), and (3) seeking help for substance use concerns. Exclusion criteria were: (1) acute intoxication or withdrawal, (2) active psychosis, (3) acute suicidality, (4) history of severe head injury, dementia, or severe neurodevelopmental disorder, and (5) other medical conditions or medications that could severely impair cognition. Additional CDiA exclusion criteria for individuals who consented to the MRI component of the study were the presence of MRI-incompatible metal implants, history of stroke, and claustrophobia.

### 2.3 Study measures

Illness awareness in AUD was measured using the Alcohol Use Awareness and Insight Scale (AAS) (25), while illness awareness in SUD was measured using the Substance Use Awareness and Insight Scale (SAS) (26). Both the AAS and SAS are self-report measures that have been developed and psychometrically tested to measure the four core domains of illness awareness: general illness awareness, symptom awareness, awareness of need for treatment, and awareness of negative consequences.^1^ Illness awareness in relation to the brain imaging analyses was assessed based on paradigm accuracy, which was calculated as the percentage of correct responses (i.e., responses consistent with intact illness awareness) on illness-related items (i.e., general illness awareness, symptom awareness, and awareness of need for treatment). To ensure equal distribution of participants between groups, those with impaired illness awareness were defined as having ≤ 77% accuracy for illness-related stimuli (*n* = 10), while those with intact illness awareness were defined as having ≥ 77% illness-related stimuli accuracy (*n* = 10).

The secondary measures of illness awareness were the ‘Recognition’ subscale of the Stages of Change Readiness and Treatment Eagerness Scale Personal Drinking Questionnaire (SOCRATES 8A) (36) and Personal Drug Use Questionnaire (SOCRATES 8D) (36). These subscales evaluate individuals’ acknowledgment of problematic substance use and their readiness to initiate change.

Severity of substance use and its associated harms were assessed using the Alcohol Use Disorder Identification Test (AUDIT) (37), Drug Use Disorder Identification Test (DUDIT) (38), and Severity of Dependence Scale (SDS) (39). Other clinical assessments included: the Timeline Followback (TLFB), the Patient Health Questionnaire-9 (PHQ-9) (40), Generalized Anxiety Disorder – 7 (GAD-7) (41), the World Health Organization Disability Assessment Schedule 2.0 (WHODAS 2.0) (42), and the World Health Organization Quality of Life Scale – Brief Version (WHOQOL-BREF) (43). Participant demographic information was collected. Other measures that are not included in this study can be referenced in the CDiA protocol (34).

### 2.4 MRI data acquisition

The MRI was performed with a 3 T GE scanner (General Electric, Waukesha, WI) equipped with a standard 32-channel head coil from Nova Medical at CAMH. Head motion was minimized by mounting foam padding equally around the head-coil. For localization purposes, high-resolution T1-weighted MPRAGE anatomical images (208 contiguous axial 1-mm-thick slices) were acquired (TR = 2,500 ms; TE = 2 ms; flip-angle 8°; 256 × 256 matrix; FOV = 25.6 cm) to assess brain structure.

#### 2.4.1 Functional imaging

The fMRI scans consisted of a BOLD-sensitive pulse sequence with whole-brain coverage. For the functional imaging session, 619 volumes (60 contiguous axial 2.4 mm thick slices) covering the whole brain were acquired using a T2*-sensitive gradient echo planar imaging (EPI) sequence (TR = 800 ms; TE = 30 ms; flip-angle 52°; 64 × 64 matrix; FOV = 21 cm). Additionally, topup scans comprising a pair of functional imaging scans with opposing phase-encoding directions of anterior-to-posterior (AP) and posterior-to-anterior (PA) were obtained prior to the illness awareness functional scan. Each scan (AP and PA) included two volumes (60 contiguous axial slices, 2.4 mm thickness) acquired in an interleaved, bottom-up order, covering the whole brain (TR = 7,400 ms, TE = 80 ms, flip-angle 90°, matrix = 90 × 90, and FOV = 21.6 cm). These scans were optimized for susceptibility distortion field estimation near air-tissue interfaces and were used to generate corrected images using FSL’s Topup tool (44).

#### 2.4.2 Illness awareness task

The MRI component for the CDiA study was approximately 1.5 h and included several tasks. The only task related to and analyzed in this study was the illness awareness paradigm designed to assess participants’ subjective illness beliefs about their substance use (Supplementary Figure 1). The illness awareness task was not part of the original CDiA protocol (*n* > 400) and was administered solely for the purposes of this pilot study in a subset of participants (*n* = 20). A similar paradigm has been employed to explore the functional neuroanatomy of impaired illness awareness in other conditions in previous studies by our group (13, 32, 45) and in another article submitted for this special issue. The illness awareness task consisted of a bank of 80 yes/agree and no/disagree statements/questions derived from four categories: general illness awareness (20 items), symptom awareness (20 items), awareness of need for treatment (20 items), and control (20 items). Approximately 75% of the items (i.e., control, general illness awareness, and need for treatment awareness items) were similar across participants, while the remaining 25% (i.e., symptom awareness items) were symptom-specific items that were tailored beforehand to match participant’s symptoms/experiences identified from the AAS/SAS.

Prior to scanning, participants completed practice items on a laptop using the E-Prime software (Psychology Software Tools, Pittsburgh, PA) (46) to confirm their understanding of the task and to ensure the symptom-specific content was relevant to their experiences. Participants were outfitted with an MR-compatible button-box during the MRI, which they used to respond to the stimuli. An adjustable mirror situated above the participant’s eyes was used to view the stimuli projected onto a screen placed at the head of the bed. Each statement was presented for 4 s, with a variable interstimulus interval of 2 s on average with a fixation cross. Participants were able to respond for up to 5 s following the presentation of the stimulus. Sample control and illness awareness questions/statements in the paradigm can be found in the Supplementary Figure 1.

### 2.5 Image preprocessing

#### 2.5.1 Functional images

The data were preprocessed and analyzed using SPM12 (47). Anatomical and functional images were reoriented to the Montreal Neurological Institute (MNI) brain template to ensure proper alignment. Skull-stripping was performed using FMRIB Software Library (FSL) (48).

To correct for susceptibility-induced distortions in the functional scan, the AP and PA topup scans were merged and processed using FSL’s topup tool to estimate the susceptibility distortion field. This estimated field was then applied to the functional images to generate distortion-corrected data in native functional space. To maintain alignment with the original acquisition space, all transformations were restricted to the native functional space. The first three of the 619 volumes were discarded for T1-equilibrium effects, and data from the remaining 616 volumes were used in preprocessing steps and analysis.

For preprocessing, the images were first slice-timing adjusted with the 20^th^ slice as the reference, then realigned to the first volume using a six-parameter rigid body transformation. Participants with head motion exceeding two voxels in rotation and translation were excluded (49, 50). The generated mean realigned functional images were coregistered with each participant’s corresponding T1-weighted structural scan, which was later segmented into six tissue maps based on SPM12 templates. Following this, the mean image was spatially normalized to the standard stereotactic space using the MNI echo planar imaging (EPI) template. The derived transformation parameters were then applied to all functional images, resampled to 3-mm^2^ isotropic voxels interpolation. Finally, the images were smoothed with a 6-mm full-width at half-maximum isotropic Gaussian kernel.

#### 2.5.2 First-level analyses

Similar to a previous study (32), first-level contrasts were created using random-effect analyses between illness-related stimuli (i.e., general illness awareness, symptom awareness, and awareness need for treatment) and control stimuli as well as between each illness awareness subdomain and control stimuli. These contrast images were subsequently entered into second-level whole-brain analyses.

#### 2.5.3 Whole brain analyses

Whole brain analysis was conducted using SPM 12 (47). Regression analyses were performed using participants’ illness awareness paradigm accuracy as the covariate of interest. Two-sample t-tests were also conducted to examine differences in BOLD responses between participants with impaired and intact illness awareness.

Separate analyses were conducted for 3 groups: (i) AUD and SUD participants combined, (ii) AUD participant group only, and (iii) SUD participant group only. Analyses for all three groups were conducted with age and gender as covariates. The AUD and SUD combined group analysis was repeated twice: (i) adding substance category (alcohol or substance) as an additional covariate, and subsequently (ii) adding illness severity (AUDIT/DUDIT scores) as an additional covariate due to the group difference in illness severity (See Table 1). One participant belonging to the SUD and intact illness awareness groups did not have a DUDIT score, so they were given the average DUDIT score to ensure their inclusion in the analysis. The analysis for the AUD only and SUD only subgroups were also repeated adding illness severity scores (AUDIT for AUD only and DUDIT for SUD only) as an additional covariate.

**Table 1:** Demographic and clinical characteristics.

	Total sample	Group comparison			
		Impaired	Intact	*t* value	*p* value
	Mean (SD)	Mean (SD)	Mean (SD)		
*n*	20	10	10	–	–
Age	35.10 (10.59)	33.30 (11.93)	36.90 (9.35)	−0.75	0.463
Gender (Female: Male)	4:16	2:8	2:8	0.00	1.000
Overall paradigm accuracy, %	78.96 (12.01)	70.02 (10.47)	87.90 (4.15)	−5.02	<0.001*
Illness Awareness paradigm accuracy	72.83 (16.19)	61.04 (14.67)	84.62 (5.44)	−4.77	<0.001*
General illness awareness stimuli	78.49 (30.00)	59.03 (32.35)	97.94 (3.55)	−3.78	0.004*
Symptom awareness stimuli	56.05 (19.04)	45.82 (17.94)	66.29 (14.52)	−2.81	0.012*
Need for treatment stimuli	84.14 (14.93)	77.18 (18.10)	91.10 (5.92)	−2.31	0.041*
Control stimuli	96.22 (3.21)	96.00 (3.16)	96.45 (3.40)	−0.31	0.764
AAS + SAS Average score	7.49 (1.74)	6.44 (1.57)	8.54 (1.21)	−3.37	0.004*
AAS Average score	7.70 (1.94)	6.76 (1.84)	8.64 (1.67)	−1.68	0.131
General illness awareness	8.50 (1.58)	8.10 (2.01)	8.90 (1.08)	−0.78	0.463
Symptom awareness	6.60 (3.13)	5.00 (2.55)	8.20 (3.03)	−1.81	0.110
Need for treatment	8.10 (1.96)	7.53 (2.42)	8.67 (1.41)	−0.90	0.399
Negative consequences	7.60 (3.53)	6.40 (4.16)	8.80 (2.68)	−1.08	0.315
SAS Average score	7.28 (1.58)	6.12 (1.38)	8.43 (0.60)	−3.45	0.016*
General illness awareness	7.00 (1.41)	6.40 (1.39)	7.60 (1.29)	−1.41	0.195
Symptom awareness	7.00 (2.49)	5.80 (2.95)	8.20 (1.30)	−1.66	0.152
Need for treatment	7.50 (1.76)	6.47 (1.57)	8.53 (1.35)	−2.23	0.057
Negative consequences	7.60 (2.76)	5.80 (2.86)	9.40 (0.89)	−2.68	0.046*
SDS – Alcohol^a^	8.42 (5.62)	8.80 (5.71)	8.00 (5.83)	0.30	0.767
SDS – Substances^b^	8.17 (5.08)	6.75 (3.96)	9.44 (5.83)	−1.13	0.279
SOCRATES 8A + SOCRATES 8D Recognition Subscale^c^	29.58 (5.70)	26.44 (5.50)	32.40 (4.40)	−2.59	0.020*
AUDIT + DUDIT^d^	23.95 (9.74)	18.80 (8.61)	29.67 (7.76)	−2.89	0.010*
TLFB	15.10 (20.99)	9.20 (16.02)	21.00 (24.41)	−1.28	0.220
PHQ-9^e^	13.47 (7.60)	13.0 (6.5)	13.9 (8.8)	−0.26	0.802
GAD-7	10.55 (7.13)	9.7 (7.8)	11.4 (6.7)	−0.52	0.607
WHODAS 2.0	81.40 (23.31)	84.00 (21.88)	78.80 (25.56)	0.49	0.631
WHOQOL-BREF	283.80 (68.52)	264.40 (63.15)	303.20 (71.33)	−1.29	0.214

AAS, Alcohol Use Awareness and Insight Scale; SAS, Substance Use Awareness and Insight Scale; SDS – Alcohol, Severity of Dependence Scale – Alcohol; SDS – Substance, Severity of Dependence Scale – Substance; SOCRATES 8A, Stages of Change Readiness and Treatment Eagerness Scale – Personal Drinking Questionnaire; SOCRATES 8D, Stages of Change Readiness and Treatment Eagerness Scale – Personal Drug Questionnaire; AUDIT, Alcohol Use Disorder Identification Test; DUDIT, Drug Use Disorder Identification Test; TLFB, Timeline Follow Back; PHQ-9, Patient Health Questionnaire-9; GAD-7, Generalized Anxiety Disorder-7; WHODAS 2.0, World Health Organization Disability Assessment Schedule 2.0; WHOQOL-BREF, World Health Organization Quality of Life Scale – Brief Version. ^a^*n* = 19 (impaired illness awareness subgroup *n* = 9). ^b^*n* = 17 (impaired illness awareness subgroup *n* = 8, intact illness awareness subgroup *n* = 9). ^c^*n* = 19 (impaired illness awareness subgroup *n* = 9). ^d^*n* = 19 (intact illness awareness subgroup *n* = 9). ^e^*n* = 19 (impaired illness awareness subgroup *n* = 9). *p < 0.05.

As the *a priori* hypothesis was specific to the PPA (19, 20, 29, 32), dorsolateral prefrontal cortex (dlPFC) (19, 29), and insula (19, 20, 29–31), this study confined the statistical search to these regions of interest (ROIs) using masks from the Automated anatomical labeling (AAL) atlas (51): bilateral angular gyri, inferior parietal regions, supramarginal gyri, superior parietal regions, and insulae. Another six *a priori* ROIs were chosen in these regions based on findings from prior neuroimaging studies done in our lab by confining a 10 mm sphere around identified peaks: bilateral angular gyri (±46, −70, 36) (20), (±42, −80, 30) (13), (±44, −60, 40) (32), inferior parietal regions (±36, −64, 42) (13), and the dlPFC (±27, 49, 24) (32), (±14, 34, 42) (13), using the Talairach Daemon atlas with WFU-Pickatlas software (52–54). The threshold for these a priori regions was set at *p* ≤ 0.001 level of significance (*t* > 3.69), 0 voxels. A cluster was reported as significant if the peak survived a familywise error (FWE) correction for multiple comparisons of *p* ≤ 0.05 (55). Given the exploratory nature of this pilot study, corrections for multiple comparisons across ROIs were not applied. For exploratory purposes, coordinates for peak brain activity that were associated with intact illness awareness were also reported and labeled using the AAL atlas.

### 2.6 Statistical analysis

Statistical analysis of clinical, demographic, and behavioral data for this study was carried out using the SPSS statistical software package (version 29.0.2.0) (56). Bivariate Pearson correlations were performed between illness awareness paradigm accuracy and relevant demographic and clinical variables. Independent t-tests were used for group comparisons of impaired versus intact illness awareness group. The significance level was established at *p* ≤ 0.05.

## 3 Results

### 3.1 Demographic and clinical data

Twelve patients with SUD and ten patients with AUD were recruited for this study. Two participants in the SUD group were excluded. One participant was excluded due to unreliable responses because they provided the same response on all items across two self-report scales (i.e., ‘4’ on a scale of 0 to 4 on DUDIT and ‘1’ on a scale of 1 to 5 on SOCRATES 8D) and did not complete the study’s primary illness awareness scale (SAS), leaving their data unsuitable for analysis. The second participant was excluded due to excessive head motion during the fMRI. The final sample consisted of 20 individuals with SUD (*n* = 10) or AUD (*n* = 10). While 9 of 10 participants in the SUD group identified cannabis as their primary substance use disorder, all of them met the criteria for cannabis use disorder. Some participants in the SUD group also had addictions to one or more substances from the following categories: sedatives, analgesics, and stimulants. Similarly, some participants in the AUD group also had addictions to one or more substances from the following categories: cannabis, sedatives, and stimulants. The exact number of participants having addictions to these other substances is not reported due to privacy concerns.

The clinical and demographic data, and comparison between impaired and intact illness awareness groups are reported in Table 1. All but one participant completed all of the study measures required for the imaging analyses. Specifically, they did not complete the DUDIT, which was used to co-vary for illness severity. One participant did not complete the SDS scale for alcohol, three participants did not complete the SDS scale for cannabis, one participant did not complete the SCORATES 8D scale, and one participant did not complete the PHQ-9 scale. Independent sample t-tests found that the impaired illness awareness group scored lower than the intact illness awareness group on AAS/SAS combined average (*t* = 3.37, *p* = 0.004), SAS average (*t* = 3.45, *p* = 0.016), SAS negative consequences subscale (*t* = 2.68, *p* = 0.046), SOCRATES 8A/8D illness recognition subscale combined average (*t* = 2.59, *p* = 0.020), and AUDIT/DUDIT average scores (*t* = 2.89, *p* = 0.010). Illness-related paradigm accuracy was positively correlated with pre-scan AAS/SAS combined average (*r* = 0.80, *p* < 0.001), AAS average (*r* = 0.79, *p* = 0.07), SAS average (*r* = 0.86, *p* = 0.001), SOCRATES 8A/8D illness recognition subscale combined average (*r* = 0.58, *p* = 0.009), and AUDIT/DUDIT average scores (*r* = 0.71, *p* < 0.001).

### 3.2 Whole brain analyses

The results for the regression and group analyses are presented in Tables 2, 3, and Supplementary Tables 1–4.

**Table 2:** Regional activations for the second-level contrast for AUD only and SUD only subgroups – regression.

Impaired illness awareness	Adjusted for age and gender							Adjusted for age, gender, and illness severity (i.e., AUDIT/DUDIT scores)						
	Cluster Maxima			Cluster size	*t* value	*p* value uncorrected	*p* value FWE corrected	Cluster Maxima			Cluster size	*t* value	*p* value uncorrected	*p* value FWE corrected
	x	y	z					x	y	z				
*AUD only*														
Illness-related > Control stimuli														
Nil								Nil						
General Illness awareness > Control stimuli														
Nil								Nil						
Symptom awareness > Control stimuli														
Nil								Nil						
Need for treatment > Control stimuli														
Nil								Nil						
*SUD only*														
Illness-related > Control stimuli														
Nil								Nil						
General illness awareness > Control Stimuli														
Right medial superior frontal gyrus	18	32	41	2	6.03	< 0.001	0.053	18	32	41	1	7.19	< 0.001	0.059
Symptom awareness > Control stimuli														
Nil								Nil						
Need for treatment > Control stimuli														
Nil								Nil						

Threshold: *p* < 0.001, uncorr., 0 voxels. All regions in association with impaired illness awareness were corrected for using a priori ROIs based on the AAL atlas and peaks identified in prior studies. *p < 0.05.

**Table 3:** Regional activations for the second-level contrast for AUD and SUD combined – group comparison.

Impaired > Intact	Adjusted for age and gender							Adjusted for age, gender, and substance category (i.e., alcohol and substance)							Adjusted for age, gender, substance category (i.e., alcohol and substance), and illness severity (i.e., AUDIT/DUDIT scores)						
	Cluster Maxima			Cluster size	*t* value	*p* value uncorrected	*p* value FWE corrected	Cluster Maxima			Cluster size	*t* value	*p* value uncorrected	*p* value FWE corrected	Cluster Maxima			Cluster size	*t* value	*p*valueuncorrected	*p*valueFWEcorrected
	x	y	z					x	y	z					x	y	z				
*AUD and SUD combined*																					
Illness-related > Control stimuli																					
Right insula	33	−19	17	1	3.98	0.001	0.094	33	−19	17	1	3.75	0.001	0.148	Nil						
General illness awareness > Control stimuli																					
Right insula	30	−19	17	2	4.49	< 0.001	0.045*	30	−19	17	2	4.25	< 0.001	0.075	Nil						
Left supramarginal gyrus	−63	−43	23	1	3.96	0.001	0.068	−63	−43	23	1	3.87	0.001	0.087	Nil						
Symptom awareness > Control stimuli																					
Nil								Nil							Nil						
Need for treatment > Control stimuli																					
Nil								Nil							Nil						

Threshold: *p* < 0.001, uncorr., 0 voxels. All regions in association with impaired illness awareness were corrected for using a priori ROIs based on the AAL atlas and peaks identified in prior studies. *p < 0.05.

#### 3.2.1 Regression analyses

There were no suprathreshold clusters identified for any contrasts of AUD and SUD groups combined and AUD subgroup only (Table 2).

For the SUD subgroup only, there was no suprathreshold clusters identified for the contrast illness-related > control stimuli. The results for the regression for the subdomain contrast general illness awareness > control revealed brain activation in the right medial superior frontal gyrus (±14, 34, 42) for impaired illness awareness with and without illness severity (DUDIT) as a covariate (Table 2; Supplementary Figure 2). However, neither finding survived FWE correction. There were no suprathreshold clusters identified for the subdomain contrasts symptom awareness > control or the awareness for need for treatment > control.

Exploratory analyses of peak BOLD responses associated with impaired illness awareness and intact illness awareness in regions outside of *a priori* ROIs are presented in Supplementary Tables 1–4. Although several peak activations were identified for AUD and SUD combined, none of them survived FWE correction. For the AUD only subgroup, there was no brain activity that survived FWE correction with only age and gender as covariates. Upon including illness severity (AUDIT) as a covariate, the results for the subdomain contrast awareness of need for treatment > control revealed a significant peak in left middle temporal gyrus (−60, −64, 5) in relation to intact illness awareness (*t* = 43.54, *p* = 0.003, FWE Corr., Supplementary Table 2). Several peaks were also identified for the SUD only subgroup, however, none of them survived FWE correction.

#### 3.2.2 Group comparison

For the AUD and SUD combined group comparison, when participants were divided into intact or impaired illness awareness using the 77% stimuli accuracy cut-off, there was an equal split in AUD (*n* = 10) versus SUD participants (*n* = 10) in both intact and impaired illness awareness groups (i.e., 5 and 5) for each substance category.

For the AUD and SUD combined group comparison between participants with impaired illness awareness versus intact illness awareness, brain activation was observed in the right insula for the contrast illness-related > control stimuli, however, it did not survive FWE correction either with or without substance category as a covariate. The results for the group comparison for the subdomain contrast general illness awareness > control revealed significant peak BOLD responses in (i) the right insula (*t* = 4.49, *p* = 0.045, FWE Corr., Hedge’s *g* = 1.92; Table 3; Supplementary Figure 3) and (ii) the left supramarginal gyrus that did not survive FWE correction. Activity in both these regions was observed after including the substance category as an additional covariate, however, neither association survived FWE correction (Table 3). No brain activity was seen when including illness severity scores (AUDIT/DUDIT) as an additional covariate for all contrasts (Table 3). There were no suprathreshold clusters identified for the subdomain contrasts symptom awareness > control or the awareness for need for treatment > control.

**Figure 1:**
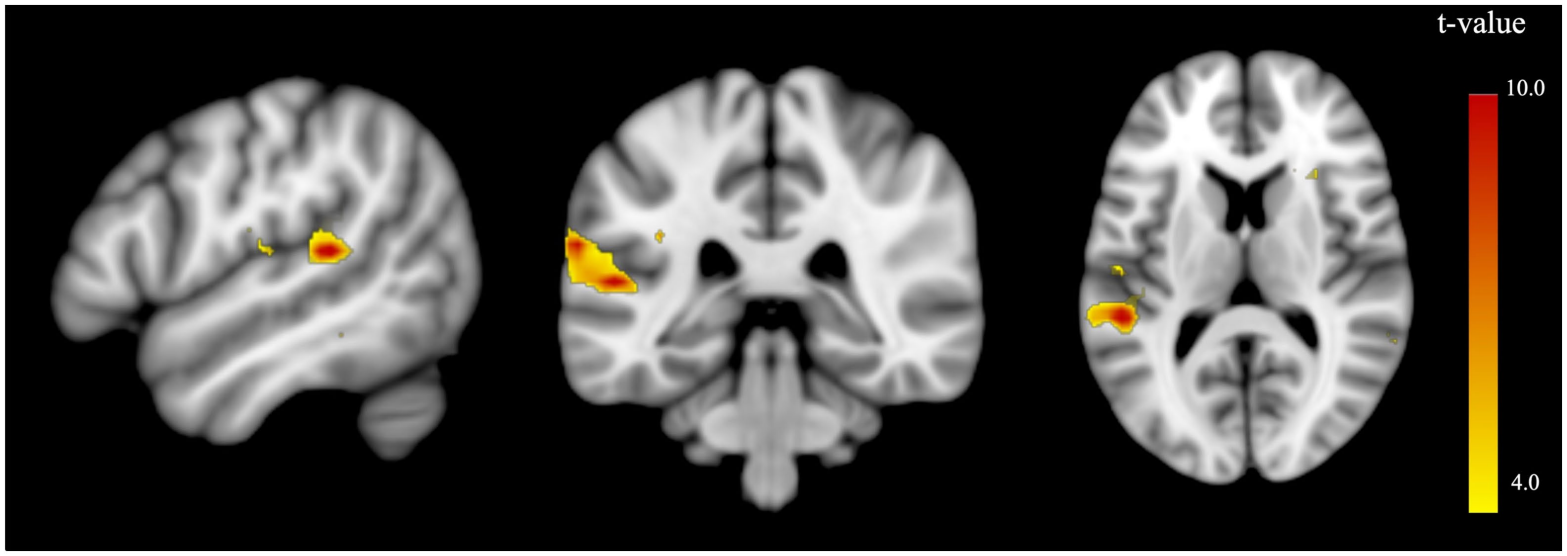
Brain activation in association with impaired illness awareness compared to intact illness awareness for the AUD subgroup for the subdomain contrast general illness awareness > control stimuli. Significant activations are presented in the left supramarginal gyrus (−63, −34, 23) (*t*=10.68, *p* = 0.007 FWE corr.). A low threshold (*p*<0.01, voxel size=0) was used to reveal all regional brain activity.

For the AUD subgroup comparison between participants with impaired illness awareness versus intact illness awareness, brain activation was observed in the left insula for the contrast illness-related > control stimuli, however, it did not survive FWE correction, nor was it observable with illness severity (AUDIT) controlled as an additional covariate (Table 4). The subdomain contrast general illness awareness > control revealed a peak BOLD response in the left supramarginal gyrus (*t* = 10.68, *p* = 0.007 FWE corr., Hedge’s *g* = 4.57; Table 4; Figure 1), while the peak did not survive FWE correction (Table 4) after including illness severity (AUDIT) as an additional covariate. There were no suprathreshold clusters identified for the subdomain contrasts symptom awareness > control or the awareness for need for treatment > control.

**Table 4:** Regional activations for the second-level contrast for AUD only and SUD only subgroups – group comparison.

Impaired > Intact	Adjusted for age and gender							Adjusted for age, gender, and illness severity (i.e., AUDIT/DUDIT scores)						
	Cluster Maxima			Cluster size	*t* value	*p* value uncorrected	*p* value FWE corrected	Cluster Maxima			Cluster size	*t* value	*p* value uncorrected	*p* value FWE corrected
	x	y	z	x	y	z
*AUD only*														
Illness-related > Control stimuli														
Left insula	−39	−1	11	1	5.93	0.001	0.174	Nil						
General illness awareness > Control stimuli														
Left supramarginal gyrus	−63	−34	23	7	10.68	< 0.001	0.007*	−63	−34	23	3	8.73	< 0.001	0.059
Symptom awareness > Control stimuli														
Nil								Nil						
Need for treatment > Control stimuli														
Nil								Nil						
*SUD only*														
Illness-related > Control stimuli														
Nil								Nil						
General illness awareness > Control stimuli														
Nil								Nil						
Symptom awareness > Control stimuli														
Nil								Nil						
Need for treatment > Control stimuli														
Nil								Nil						

Threshold: *p* < 0.001, uncorr., 0 voxels. All regions in association with impaired illness awareness were corrected for using a priori ROIs based on the AAL atlas and peaks identified in prior studies. *p < 0.05.

For the SUD subgroup comparison between participants with impaired illness awareness versus intact illness awareness, there were no suprathreshold clusters identified for any contrasts.

Exploratory analyses of peak BOLD response in regions outside of *a priori* ROIs and for comparison between intact illness awareness versus impaired illness awareness are reported in Supplementary Tables 1–4. Although several peak activations were identified, no intact illness awareness versus impaired illness awareness (i.e., intact illness awareness > impaired illness awareness) or impaired illness awareness versus intact illness awareness (i.e., impaired illness awareness > intact illness awareness) survived FWE correction.

## 4 Discussion

Impaired illness awareness is a clinically important barrier to treatment engagement and clinical outcomes in individuals with substance use disorders (SUDs) (4, 5). To our knowledge, this pilot exploratory study is among the first to investigate the neural correlates of impaired illness awareness in SUD using a comprehensive, task-based fMRI approach. This study found that individuals with SUDs with impaired illness awareness compared to intact illness awareness had higher brain activity (i.e., increased BOLD responses) in the frontoparietal and insular regions, specifically the PPA and insula, and secondarily, the right dlPFC, in relation to an fMRI illness awareness task. Although present within the *a priori* ROIs, none of these results survived correction for multiple comparisons. At the same time, they are consistent with previous findings in other conditions (13, 19, 20, 27, 29) and the results of another study presented in this issue exploring the neural correlates of impaired illness awareness in obesity. Although preliminary, this study’s findings broadly align with prior research linking frontoparietal and insular areas, regions part of the DMN and involved in self-referential processing, as being related to impaired illness awareness in SUDs (27) and other conditions that feature impaired illness awareness, such as schizophrenia and stroke (13, 19, 20, 29).

Increased BOLD activation in the left supramarginal gyrus, part of the left PPA, was observed in the group comparison analysis (i.e., impaired illness awareness > intact illness awareness) in the AUD and SUD combined group and AUD subgroup only, when controlling for age and gender. However, the peak only survived correction for multiple comparisons in the AUD only subgroup, suggesting the finding was driven by the AUD rather than the SUD subgroup. This study’s results also revealed significant BOLD response in the right insula in the AUD and SUD combined group when controlling for age and gender. Notably, all the aforementioned findings became non-significant after correction for illness severity (i.e., AUDIT/DUDIT scores), indicating that illness severity may play a moderating role in the relationship between illness awareness and brain activation. BOLD activation in the right dlPFC, specifically the medial superior frontal gyrus, was also observed in the SUD only subgroup, after controlling for age and gender, with and without controlling for illness severity. However, neither association survived correction for multiple comparisons, possibly due to the limited sample size.

Collectively, this study’s findings, while preliminary, are important as they align with the existing literature on impaired illness awareness in other disorders. Frontoparietal and insular regions are key parts of the DMN and have been consistently linked to dysfunction in self-referential processing in stroke (12, 13), Alzheimer’s disease (14, 15), schizophrenia (16–21), mood disorders (15), hypertension (22), diabetes (23), obesity (24), and addictions (25–28). For instance, prior work from group has reported increased activation in the PPA, insula, and prefrontal cortex in individuals with schizophrenia and impaired illness awareness during similar illness awareness tasks (20, 32). The few studies that have investigated the neural correlates of impaired illness awareness in SUD suggest that impaired illness awareness may be associated with differential activation in frontoparietal cortices and the insula which are linked with self-awareness and self-referential deficits (27, 28, 33).

This study’s clinical and demographic findings provide additional context for understanding illness awareness in addiction. Greater illness awareness was associated with greater severity of substance use, consistent with prior research on impaired illness awareness in SUDs (25, 26, 57, 58), obesity (59), diabetes (60), and hypertension (61). That is, the greater the severity of addiction, the more likely the individual is to gain subjective illness awareness. This suggests that greater efforts may be required clinically to promote illness recognition earlier in the illness course to prevent progression to a SUD and more serious negative clinical outcomes. Research shows that early intervention is associated with greater treatment engagement and decreased substance use and substance-related harms (62–64).

There are several limitations of this study. First, while a significant BOLD response was observed in the PPA and insula in relation to impaired illness awareness after controlling for age and gender, the activation became non-significant after controlling for illness severity. The non-significant findings after adjusting for illness severity likely reflect collinearity between illness awareness (i.e., paradigm accuracy) and illness severity rather than the absence of a true association. Further, the validity and specificity of the illness awareness task mitigates this limitation. The task elicits moment-to-moment neural responses associated with accepting versus denying illness-related statements, allowing the measurement of BOLD signal changes that correspond to self-referential processing of illness awareness. This paradigm has previously demonstrated sensitivity in distinguishing neural activity linked to impaired versus intact illness awareness in schizophrenia (13, 32). Still, findings should be treated with caution until replicated in future, more powered, studies. Second, the study only included a small, treatment-seeking sample that likely limited the variability in illness awareness and the strength of study findings. A majority of the sample demonstrated relatively high illness awareness, constraining the range and sensitivity of this study’s analyses. Third, the substance use group had significant heterogeneity in terms of substances used. While most SUD participants met diagnostic criteria for cannabis only, the remaining SUD individuals met the diagnostic criteria for sedatives, analgesics, and stimulants. Similarly, while some AUD participants met diagnostic criteria for alcohol only, the remaining six AUD individuals met diagnostic criteria for cannabis, sedatives, and stimulants. While polysubstance users may show differential activation in resting-state or metabolic imaging studies (28, 65–67), task-based fMRI paradigms, such as the one employed in this current study, help isolate task-specific neural processes, thereby minimizing the influence of polysubstance use (68–70). Moreover, as polysubstance use is highly prevalent among treatment-seeking individuals with SUDs (approximately 91% with an average of 3.5 substances), the current study’s sample reflects the real-world clinical population (71). Due to the small sample size, we could also not report the exact number of participants with addictions to other substances. The decision was made in accordance with CDiA research program guidelines developed by their Biostatistical Consulting Service and lived experience advisory committee, to balance transparency with participant privacy. Due to the small sample size, we did not report the exact number of participants with addictions to other substances. The decision was made in accordance with CDiA research program guidelines designed to balance transparency with participant privacy. In a small sample, even with de-identified data, participants’ specific substance use profiles and comorbid psychiatric diagnoses can theoretically increase the risk of identification by others or participants themselves ours (72–75). This limitation can be addressed in future studies with larger samples. Fourth, 80% of the sample was male which may have impacted generalizability of findings. To minimize the effect of gender on the findings, all results reported in this manuscript controlled for sex as a covariate. Fifth, the pilot, exploratory nature of this study limited controlling for other important factors, such as individual differences in cognitive function, substance use education, or cultural background, all of which prior research suggests may influence subjective illness awareness in SUDs. For example, poorer executive function is associated with lower illness awareness (76), while cultural constructs such as familism and fatalism may alter how symptoms are understood or reported (77).

Given this study’s limitations and the negative impact of impaired illness awareness on treatment engagement and clinical outcomes (11, 57, 78–81), further neuroimaging research prioritizing larger sample sizes to account for polysubstance use is required to identify the neural correlates of impaired illness awareness in SUD. Future studies should also examine resting state functional connectivity between frontoparietal and insular regions, as network-level interactions may shed more light on the mechanisms of substance use awareness. Once reliable brain biomarkers are established, neurostimulation techniques such as transcranial direct current and magnetic stimulation may be explored as treatments to improve illness awareness, as they are in other conditions like schizophrenia and stroke (32, 82–87).

## 5 Conclusion

The results of this pilot exploratory study found that impaired illness awareness is associated with increased activation in the left PPA and right insula during a substance use awareness task, contributing to the limited research on the neural correlates of impaired illness awareness in SUDs. While these preliminary findings did not survive correction for multiple testing, this study’s findings are valuable as they highlight a possible transdiagnostic association between frontoparietal and insular dysfunction and impaired illness awareness. These findings align with previous studies in impaired illness awareness across different clinical populations that observed frontoparietal and insular dysfunction in individuals with impaired illness awareness. However, these results in a small, heterogenous sample underscore the need for the replication of this study in larger samples to clarify the role of these brain regions and their relevance for future treatment development for impaired illness awareness in SUDs.

## Data Availability

The raw data supporting the conclusions of this article will be made available by the authors, without undue reservation.

## Footnotes

1
http://illnessawarenessscales.com/

## The group members of the CDiA program study group

CDiA Program Study Group consists of L. C. Q., E. S., D. F., S. L., T. P., Y. S. N., A. C. R., Jeffrey Wardell, E. V., D. V., Isabelle Boileau, Nikki Bozinoff, Daniel Blumberger, Christopher Bowie, Paul Fletcher, P. G., Ahmed Hassan, Colin Hawco, Yarissa Herman, Pamela Kaduri, Bernard Le Foll, Osnat Melamed, Lena Quigley, Jürgen Rehm, Kevin Shield, Matthew Sloan, Victor Tang, Wei Wang, and Samantha Wells.
