# Supplementary material for: The neural correlates of illness awareness in addiction: a pilot exploratory analysis of preliminary data from the cognitive dysfunction in the addictions (CDiA) research program
Source: Front Neurol. 2026 Jan 5;16:1694826. doi: 10.3389/fneur.2025.1694826 (PMC12812657; doi:10.3389/fneur.2025.1694826)
Supplement: Supplementary file 1 [file Supplementary_file_1.docx]

Supplementary Material

# Supplementary Figures


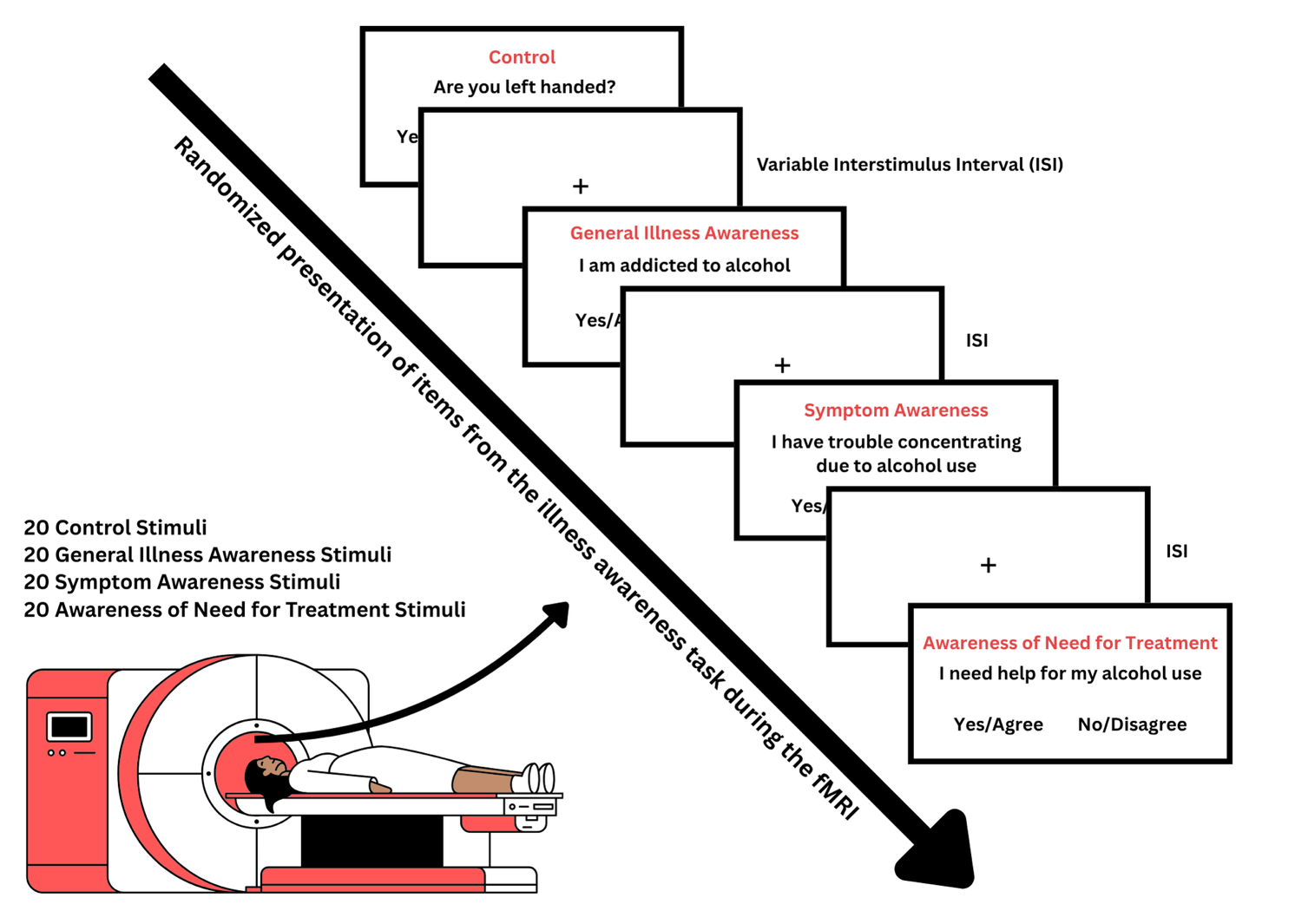
**Supplementary Figure 1. Illness Awareness Task Stimuli in Paradigm.** Functional MRI task and sample statements designed to confront participants with their beliefs about their illness. An adjustable mirror located above the participant's eyes was used to view the statements projected onto a screen placed at the head of the bed using the E-Prime software (Psychology Software Tools, Pittsburgh, PA). Each statement was presented for 4s, with a variable interstimulus interval of 2s on average, where participants viewed a fixation cross. Participants were able to respond for up to 5 seconds following the presentation of the stimulus. Each participant was outfitted with an MR-compatible button-box.

8.0

t-value

2.0


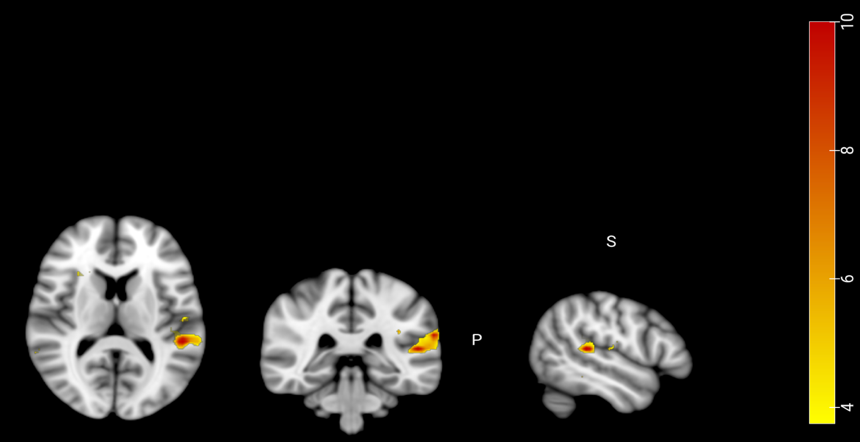

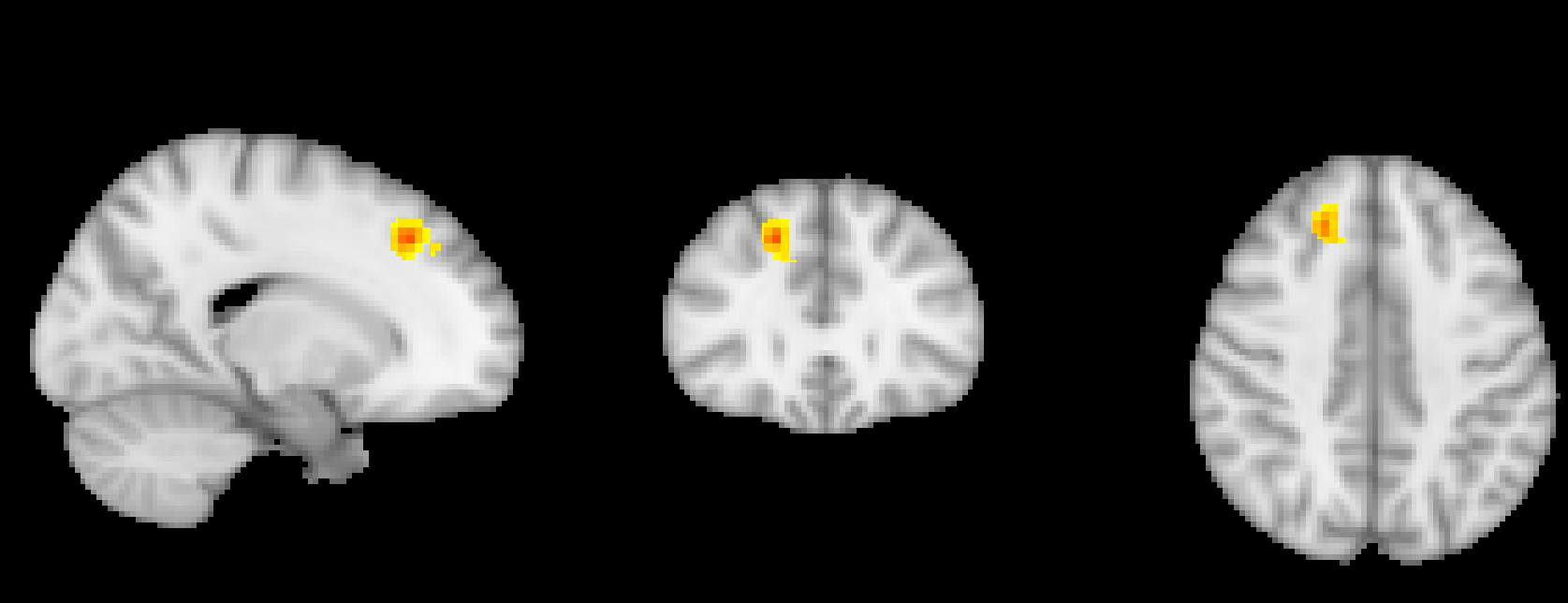

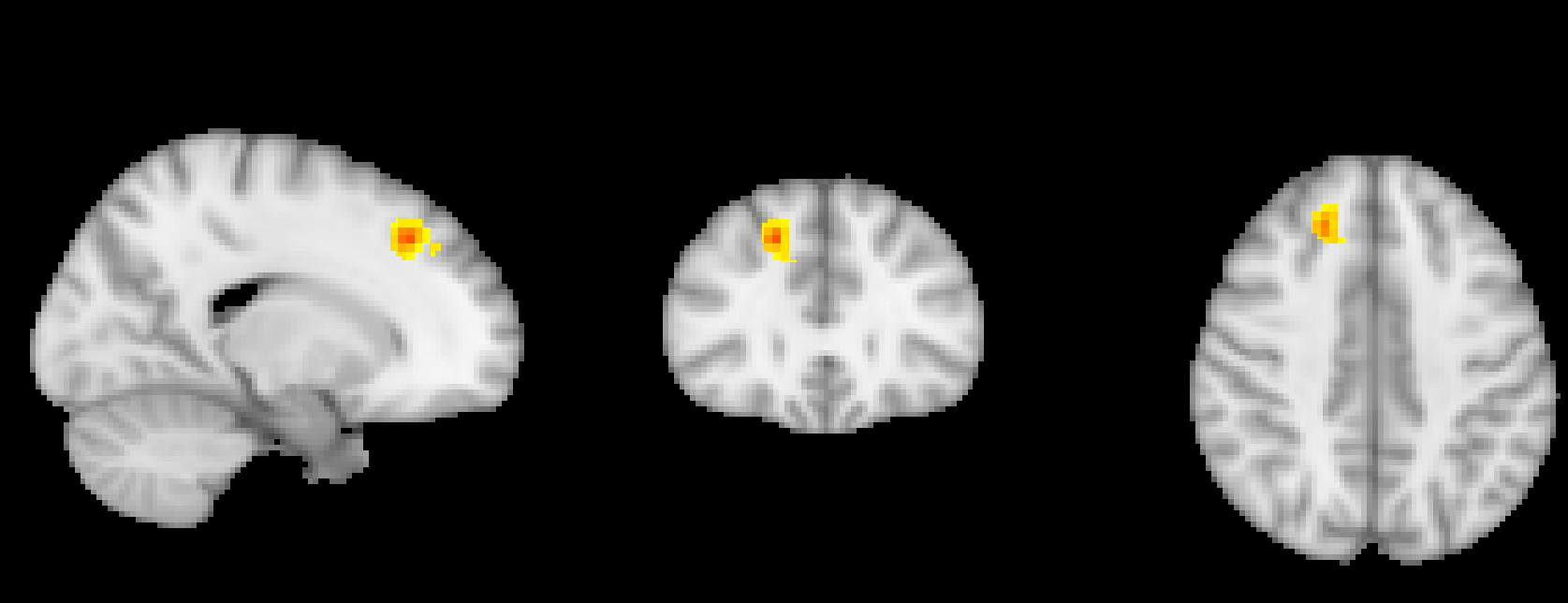

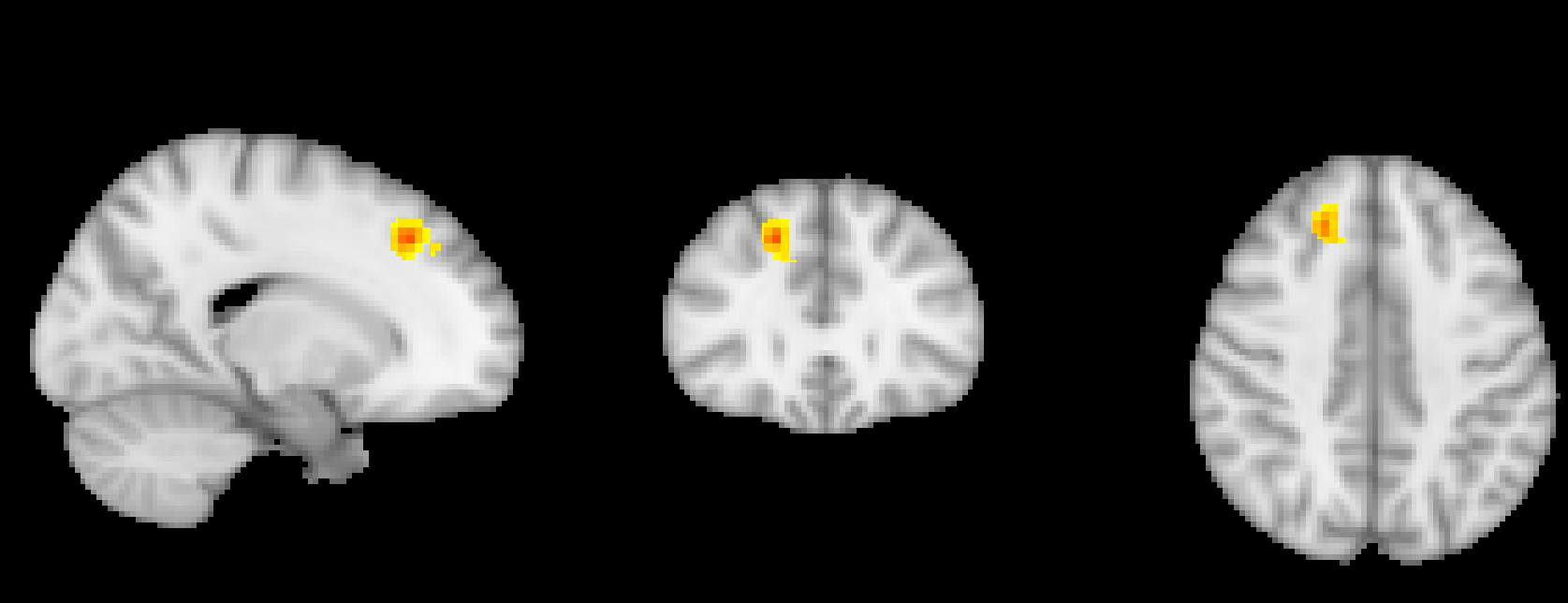


**Supplementary Figure 2.** Brain activation in association with impaired illness awareness for the SUD subgroup for the subdomain contrast general illness awareness > control stimuli. Significant activations are presented in the right medial superior frontal gyrus (±14, 34, 42) (*t* = 43.54, *p* = 0.003, FWE Corr.). A low threshold (*p* < 0.01, voxel size = 0) was used to reveal all regional brain activity.


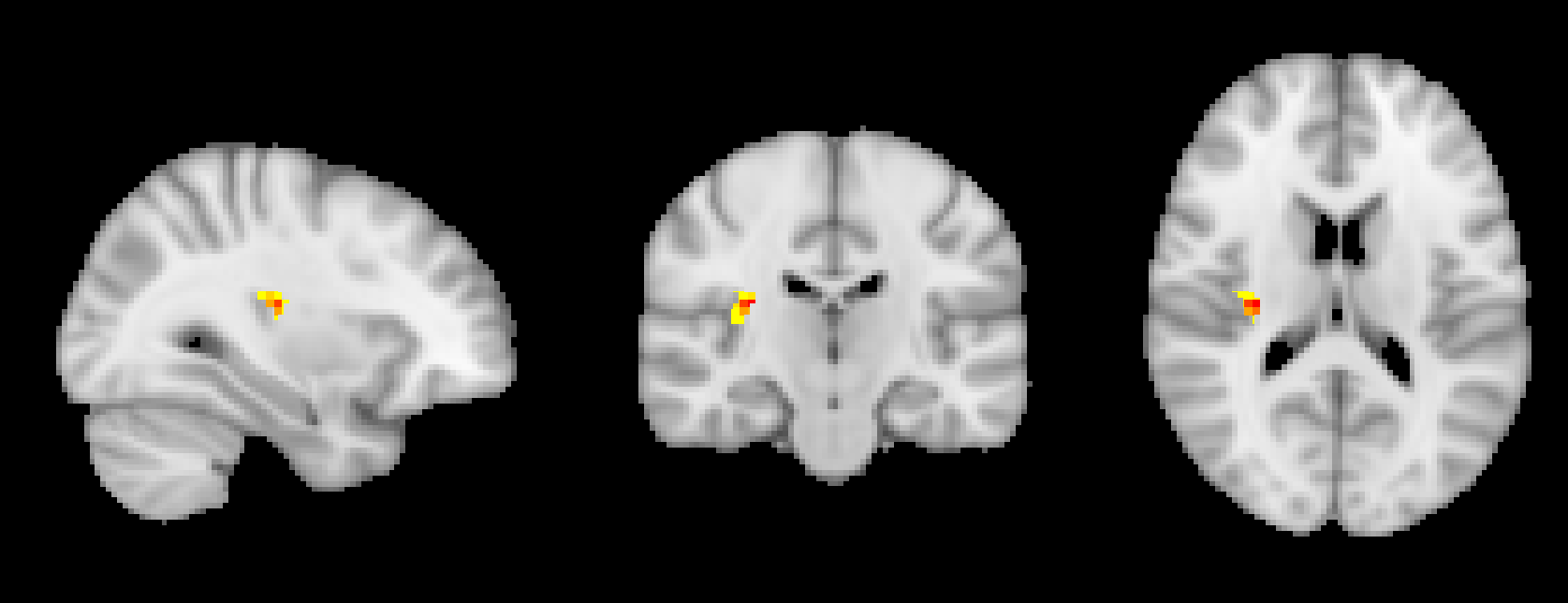

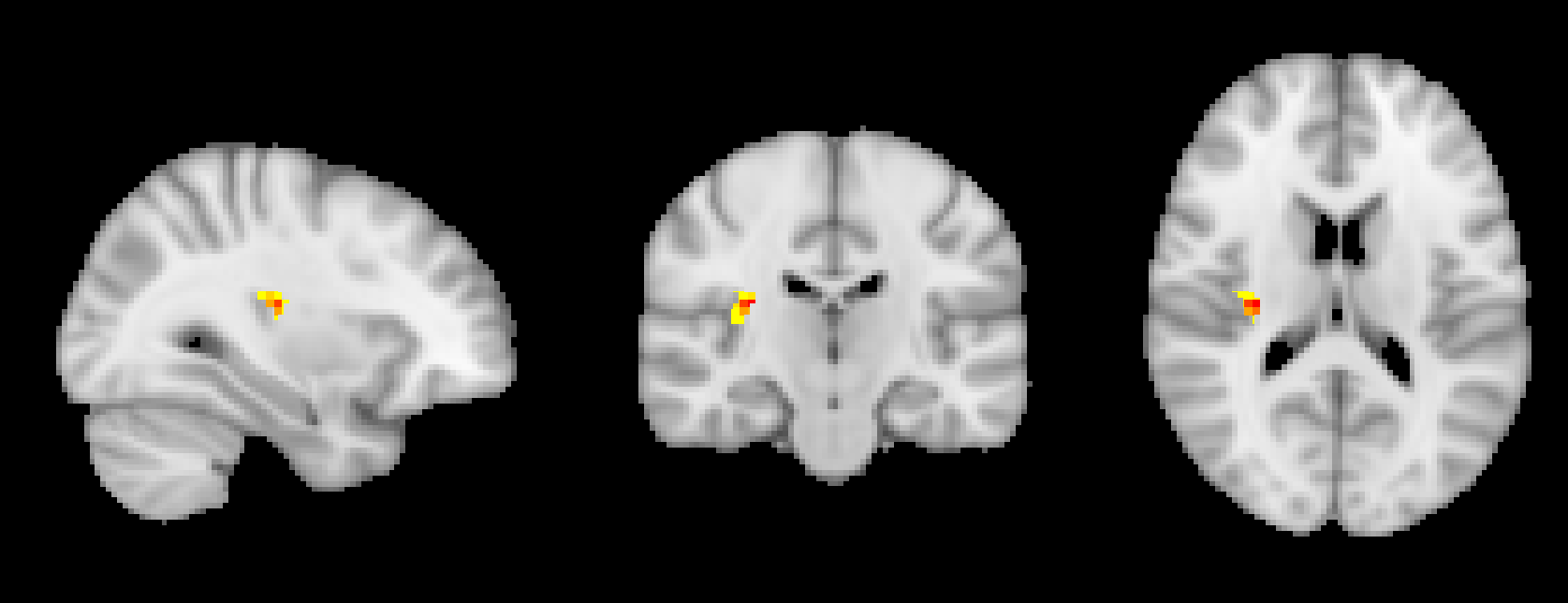

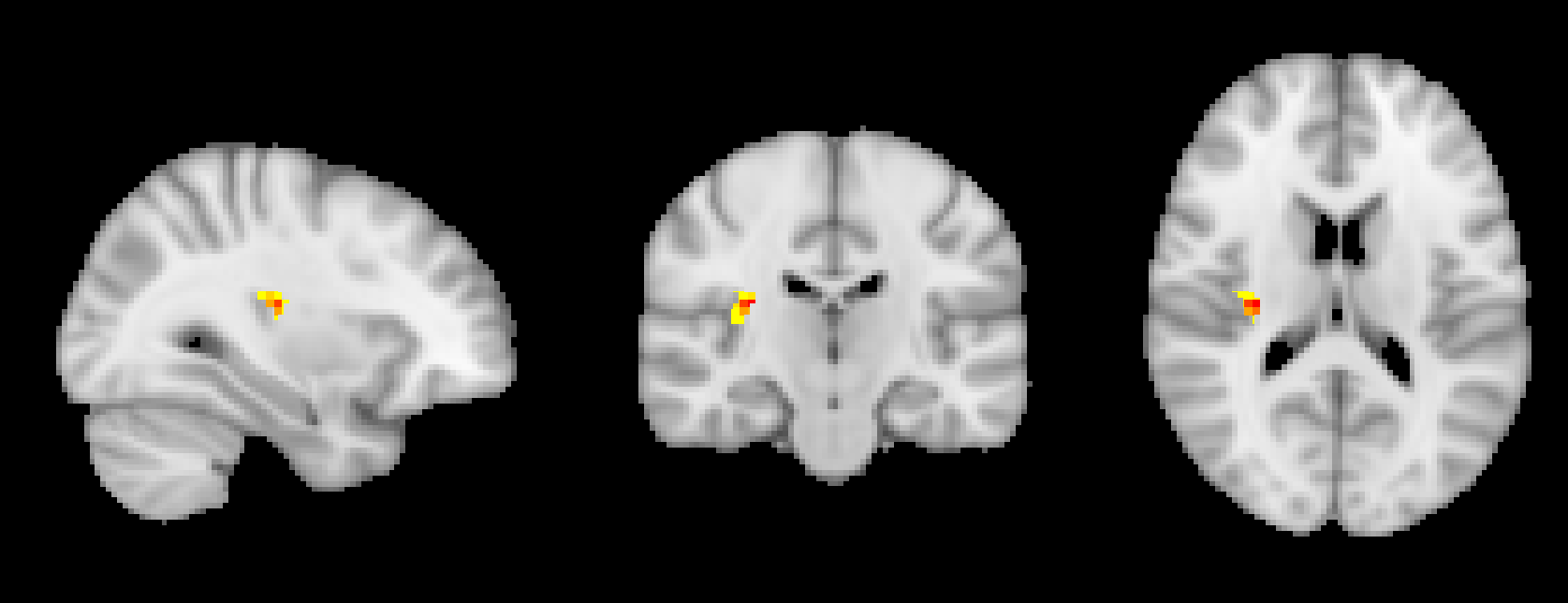


8.0

t-value

2.0


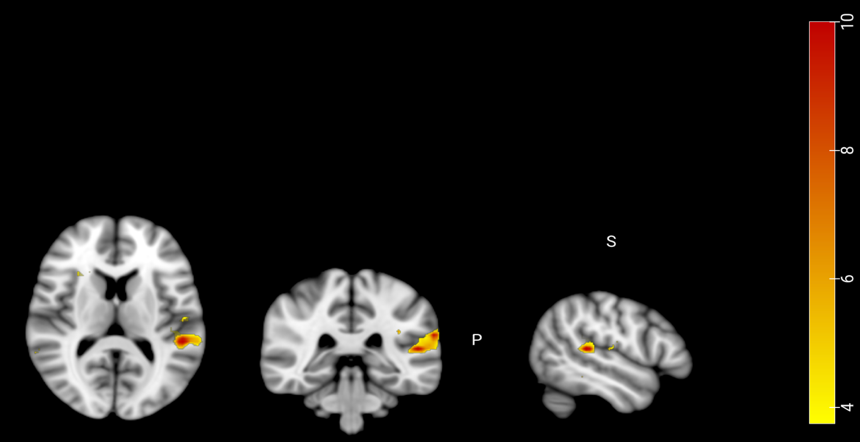


**Supplementary Figure 3.** Brain activation in association with impaired illness awareness compared to intact illness awareness for the AUD and SUD subgroups combined for the subdomain contrast general illness awareness > control stimuli. Significant activations are presented in the right insula (-30, −19, 17) (*t* = 4.49, *p* = 0.045, FWE Corr.). A low threshold (p < 0.01, voxel size = 0) was used to reveal all regional brain activity.

# Supplementary Tables

| **Supplementary Table 1. Regional activations for the second-level contrast for AUD and SUD combined – Regression** | | | | | | | | | | | | | | | | | | | | | |
| --- | --- | --- | --- | --- | --- | --- | --- | --- | --- | --- | --- | --- | --- | --- | --- | --- | --- | --- | --- | --- | --- |
|  | **Adjusted for age and gender** | | | | | | | **Adjusted for age, gender, and substance category (i.e., alcohol and substance)** | | | | | | | **Adjusted for age, gender, substance category (i.e., alcohol and substance), and illness severity (i.e., AUDIT/DUDIT scores)** | | | | | | |
|  | Cluster Maxima | | | Cluster size | *t* value | *p* value uncorrected | *p* value FWE corrected | Cluster Maxima | | | Cluster size | *t* value | *p* value uncorrected | *p* value FWE corrected | Cluster Maxima | | | Cluster size | *t* value | *p* value uncorrected | *p* value FWE corrected |
|  | x | y | z |  |  |  |  | x | y | z |  |  |  |  | x | y | z |  |  |  |  |
| **Illness-related > Control stimuli** | | | | | | | | | | | | | | |  | | | | | | |
| **Impaired** | | | | | | | | | | | | | | |  | | | | | | |
| Left putamen | Nil |  |  |  |  |  |  | -30 | -4 | 11 | 1 | 3.91 | 0.001 | 0.997 | Nil |  |  |  |  |  |  |
|  | | | | | | | | | | | | | | | | | | | | | |
| **Intact** | | | | | | | | | | | | | | | | | | | | | |
| Left precuneus | Nil |  |  |  |  |  |  | -15 | -52 | 17 | 1 | 3.88 | 0.001 | 0.998 | Nil |  |  |  |  |  |  |
| Left thalamus | Nil |  |  |  |  |  |  | Nil |  |  |  |  |  |  | -24 | -25 | 14 | 3 | 5.41 | < 0.001 | 0.634 |
| Unlabeled | Nil |  |  |  |  |  |  | Nil |  |  |  |  |  |  | 15 | -22 | -19 | 2 | 4.64 | < 0.001 | 0.935 |
| Left putamen | Nil |  |  |  |  |  |  | Nil |  |  |  |  |  |  | -21 | 2 | 11 | 3 | 4.51 | < 0.001 | 0.961 |
| Left anterior cingulum | Nil |  |  |  |  |  |  | Nil |  |  |  |  |  |  | -6 | 17 | 23 | 2 | 4.50 | < 0.001 | 0.962 |
| Left thalamus | Nil |  |  |  |  |  |  | Nil |  |  |  |  |  |  | -6 | -13 | 5 | 2 | 4.25 | < 0.001 | 0.989 |
| Right posterior cingulum | Nil |  |  |  |  |  |  | Nil |  |  |  |  |  |  | 12 | -43 | 17 | 2 | 4.17 | < 0.001 | 0.993 |
| Right inferior occipital gyrus | Nil |  |  |  |  |  |  | Nil |  |  |  |  |  |  | 42 | -76 | -7 | 1 | 4.14 | 0.001 | 0.994 |
| Left cuneus | Nil |  |  |  |  |  |  | Nil |  |  |  |  |  |  | -15 | -49 | 23 | 2 | 4.11 | 0.001 | 0.996 |
| Left thalamus | Nil |  |  |  |  |  |  | Nil |  |  |  |  |  |  | -9 | -19 | 5 | 2 | 4.10 | 0.001 | 0.996 |
| Left operculum of the inferior frontal gyrus | Nil |  |  |  |  |  |  | Nil |  |  |  |  |  |  | 54 | 11 | 5 | 1 | 3.94 | 0.001 | 0.999 |
| Left thalamus | Nil |  |  |  |  |  |  | Nil |  |  |  |  |  |  | -18 | -19 | 8 | 1 | 3.82 | 0.001 | 1.000 |
| Left cerebellum crus III | Nil |  |  |  |  |  |  | Nil |  |  |  |  |  |  | -9 | -31 | -16 | 1 | 3.81 | 0.001 | 1.000 |
| Right anterior cingulum | Nil |  |  |  |  |  |  | Nil |  |  |  |  |  |  | 15 | 41 | 11 | 1 | 3.81 | 0.001 | 1.000 |
| **General illness awareness > Control stimuli** | | | | | | | | | | | | | | | | | | | | | |
| **Impaired** | | | | | | | | | | | | | | | | | | | | | |
| Right inferior frontal gyrus (triangular part) | 51 | 29 | 32 | 1 | 4.15 | < 0.001 | 0.980 | 51 | 29 | 32 | 1 | 4.00 | 0.001 | 0.996 | Nil |  |  |  |  |  |  |
|  | | | | | | | | | | | | | | | | | | | | | |
| **Intact** | | | | | | | | | | | | | | | | | | | | | |
| Left supplementary motor area | Nil |  |  |  |  |  |  | Nil |  |  |  |  |  |  | -24 | -25 | 14 | 3 | 5.39 | < 0.001 | 0.658 |
| Right inferior occipital gyrus | Nil |  |  |  |  |  |  | Nil |  |  |  |  |  |  | 39 | -76 | -7 | 10 | 5.05 | < 0.001 | 0.811 |
| Left cerebellum crus IV and V | Nil |  |  |  |  |  |  | Nil |  |  |  |  |  |  | -18 | -43 | -28 | 2 | 4.12 | 0.001 | 0.996 |
| Right middle temporal gyrus | Nil |  |  |  |  |  |  | Nil |  |  |  |  |  |  | 51 | -55 | 2 | 1 | 4.05 | 0.001 | 0.997 |
| Right parahippocamal area | Nil |  |  |  |  |  |  | Nil |  |  |  |  |  |  | 15 | -25 | -19 | 1 | 4.03 | 0.001 | 0.998 |
| Left supplementary motor area | Nil |  |  |  |  |  |  | Nil |  |  |  |  |  |  | -6 | 23 | 62 | 2 | 3.92 | 0.001 | 0.999 |
|  | | | | | | | | | | | | | | | | | | | | | |
| **Symptom awareness > Control stimuli** | | | | | | | | | | | | | | | | | | | | | |
| **Impaired** | | | | | | | | | | | | | | | | | | | | | |
|  | Nil |  |  |  |  |  |  | Nil |  |  |  |  |  |  | Nil |  |  |  |  |  |  |
|  | | | | | | | | | | | | | | | | | | | | | |
| **Intact** | | | | | | | | | | | | | | | | | | | | | |
| Right precuneus | 12 | -46 | 20 | 1 | 3.91 | 0.001 | 0.995 | 12 | -46 | 20 | 1 | 4.08 | < 0.001 | 0.991 | Nil |  |  |  |  |  |  |
| Right putamen | Nil |  |  |  |  |  |  | 24 | 23 | -4 | 1 | 3.90 | 0.001 | 0.997 | Nil |  |  |  |  |  |  |
| Left thalamus | Nil |  |  |  |  |  |  | Nil |  |  |  |  |  |  | -24 | -25 | 14 | 3 | 5.51 | < 0.001 | 0.596 |
| Left superior temporal gyrus | Nil |  |  |  |  |  |  | Nil |  |  |  |  |  |  | -42 | -22 | -1 | 6 | 5.35 | < 0.001 | 0.669 |
| Left supplementary motor area | Nil |  |  |  |  |  |  | Nil |  |  |  |  |  |  | -6 | 5 | 50 | 5 | 4.63 | < 0.001 | 0.939 |
| Right supramarginal gyrus | Nil |  |  |  |  |  |  | Nil |  |  |  |  |  |  | 66 | -25 | 23 | 3 | 4.57 | < 0.001 | 0.952 |
| Right posterior cingulum | Nil |  |  |  |  |  |  | Nil |  |  |  |  |  |  | 12 | -43 | 17 | 5 | 4.35 | < 0.001 | 0.982 |
| Right middle temporal gyrus | Nil |  |  |  |  |  |  | Nil |  |  |  |  |  |  | 51 | -73 | 2 | 6 | 4.28 | < 0.001 | 0.988 |
| Right operculum of the rolandic gyrus | Nil |  |  |  |  |  |  | Nil |  |  |  |  |  |  | 51 | 5 | 8 | 5 | 4.27 | < 0.001 | 0.988 |
| Right inferior temporal gyrus | Nil |  |  |  |  |  |  | Nil |  |  |  |  |  |  | 57 | -67 | -7 | 5 | 4.23 | < 0.001 | 0.991 |
| Left thalamus | Nil |  |  |  |  |  |  | Nil |  |  |  |  |  |  | -15 | -16 | 8 | 8 | 4.22 | < 0.001 | 0.991 |
| Left anterior cingulum | Nil |  |  |  |  |  |  | Nil |  |  |  |  |  |  | -6 | 14 | 23 | 6 | 4.14 | < 0.001 | 0.995 |
| Left inferior parietal gyrus | Nil |  |  |  |  |  |  | Nil |  |  |  |  |  |  | -57 | -31 | 41 | 5 | 4.08 | 0.001 | 0.997 |
| Left middle occipital gyrus | Nil |  |  |  |  |  |  | Nil |  |  |  |  |  |  | -54 | -73 | 5 | 2 | 4.06 | 0.001 | 0.997 |
| Right anterior cingulum | Nil |  |  |  |  |  |  | Nil |  |  |  |  |  |  | 6 | 8 | 26 | 1 | 3.97 | 0.001 | 0.999 |
| Left middle temporal gyrus | Nil |  |  |  |  |  |  | Nil |  |  |  |  |  |  | -66 | -37 | 8 | 2 | 3.96 | 0.001 | 0.999 |
| Right precuneus | Nil |  |  |  |  |  |  | Nil |  |  |  |  |  |  | 9 | -43 | 56 | 1 | 3.90 | 0.001 | 0.999 |
| Right caudate | Nil |  |  |  |  |  |  | Nil |  |  |  |  |  |  | 9 | 11 | 20 | 1 | 3.89 | 0.001 | 0.999 |
| Left middle temporal gyrus | Nil |  |  |  |  |  |  | Nil |  |  |  |  |  |  | -60 | -64 | 5 | 1 | 3.84 | 0.001 | 1.000 |
| Right cerebellum crus VI | Nil |  |  |  |  |  |  | Nil |  |  |  |  |  |  | 30 | -70 | -22 | 1 | 3.81 | 0.001 | 1.000 |
| Right superior frontal gyrus | Nil |  |  |  |  |  |  | Nil |  |  |  |  |  |  | 21 | -1 | 53 | 1 | 3.80 | 0.001 | 1.000 |
|  | | | | | | | | | | | | | | | | | | | | | |
|  | | | | | | | | | | | | | | | | | | | | | |
| **Need for treatment > Control stimuli** | | | | | | | | | | | | | | | | | | | | | |
| **Impaired** | | | | | | | | | | | | | | | | | | | | | |
| Unlabeled | Nil |  |  |  |  |  |  | -24 | -13 | 20 | 2 | 4.01 | 0.001 | 0.994 | Nil |  |  |  |  |  |  |
|  | | | | | | | | | | | | | | | | | | | | | |
| **Intact** | | | | | | | | | | | | | | | | | | | | | |
| Left fusiform gyrus | -27 | -19 | -28 | 2 | 4.83 | < 0.001 | 0.744 | -27 | -19 | -28 | 2 | 4.64 | < 0.001 | 0.887 |  |  |  |  |  |  |  |
| Right Anterior Cingulum | 12 | 35 | 20 | 1 | 3.77 | 0.001 | 0.998 | Nil |  |  |  |  |  |  | Nil |  |  |  |  |  |  |
| Left thalamus | Nil |  |  |  |  |  |  | Nil |  |  |  |  |  |  | -3 | -13 | 5 | 14 | 6.13 | < 0.001 | 0.334 |
| Left inferior frontal gyrus (triangular part) | Nil |  |  |  |  |  |  | Nil |  |  |  |  |  |  | -33 | 29 | -1 | 4 | 5.40 | < 0.001 | 0.635 |
| Left anterior cingulum | Nil |  |  |  |  |  |  | Nil |  |  |  |  |  |  | -6 | 17 | 23 | 4 | 4.59 | < 0.001 | 0.943 |
| Left putamen | Nil |  |  |  |  |  |  | Nil |  |  |  |  |  |  | -21 | 2 | 8 | 3 | 4.11 | 0.001 | 0.995 |
| Left anterior cingulum | Nil |  |  |  |  |  |  | Nil |  |  |  |  |  |  | -3 | 35 | -1 | 1 | 3.98 | 0.001 | 0.998 |
| Right middle cingulum | Nil |  |  |  |  |  |  | Nil |  |  |  |  |  |  | 6 | -16 | 35 | 1 | 3.96 | 0.001 | 0.998 |
| Left Helsch’s gyrus | Nil |  |  |  |  |  |  | Nil |  |  |  |  |  |  | -36 | -28 | 5 | 1 | 3.95 | 0.001 | 0.998 |
| Left middle frontal gyrus | Nil |  |  |  |  |  |  | Nil |  |  |  |  |  |  | -42 | 41 | 17 | 1 | 3.95 | 0.001 | 0.999 |
| Right parahippocampal area | Nil |  |  |  |  |  |  | Nil |  |  |  |  |  |  | 15 | -31 | -10 | 1 | 3.80 | 0.001 | 1.000 |
| Right operculum of the rolandic gyrus | Nil |  |  |  |  |  |  | Nil |  |  |  |  |  |  | 48 | -16 | 14 | 1 | 3.79 | 0.001 | 1.000 |
| Note: Threshold: *p* < 0.001, uncorr., 0 voxels;  **p* < 0.05 FWE corrected | | | | | | | | | | | | | | | | | | | | | |

| **Supplementary Table 2. Regional activations for the second-level contrast for AUD only and SUD only groups – Regression** | | | | | | | | | | | | | | | | | |
| --- | --- | --- | --- | --- | --- | --- | --- | --- | --- | --- | --- | --- | --- | --- | --- | --- | --- |
|  | **Adjusted for age and gender** | | | | | | | | **Adjusted for age, gender, and illness severity (i.e., AUDIT/DUDIT scores)** | | | | | | | | |
|  | Cluster Maxima | | | Cluster size | *t* value | | *p* value uncorrected | *p* value FWE corrected | Cluster Maxima | | | | | Cluster size | *t* value | *p* value uncorrected | *p* value FWE corrected |
|  | x | y | z |  |  | |  |  | x | y | | z | |  |  |  |  |
| ***AUD subgroup only*** | | | | | | | | | | | | | | | | | |
| **Illness-related > Control stimuli** | | | | | | | | | | | | | | | | | |
| **Impaired** | | | | | | | | | | | | | | | | | |
| Nil | | | | | | | | | Nil |  |  | |  | |  |  |  |
|  | | | | | | | | | | | | | | | | | |
| **Intact** | | | | | | | | | | | | | | | | | |
| Right middle frontal gyrus | 39 | 38 | 38 | 6 | 8.78 | | < 0.001 | 0.985 | 39 | 38 | 38 | | 1 | | 6.34 | 0.001 | 1.000 |
| Left precuneus | -15 | -52 | 17 | 1 | 6.55 | | < 0.001 | 1.000 | -15 | -52 | 17 | | 2 | | 11.73 | < 0.001 | 0.998 |
| Left superior temporal gyrus | -48 | -13 | -1 | 1 | 6.43 | | < 0.001 | 1.000 | -48 | -13 | -1 | | 2 | | 6.03 | 0.001 | 1.000 |
| Right superior orbital frontal gyrus | 15 | 23 | -16 | 2 | 6.12 | | < 0.001 | 1.000 | Nil |  |  | |  | |  |  |  |
| Right medial superior frontal gyrus | 9 | 50 | 2 | 1 | 5.92 | | 0.001 | 1.000 | Nil |  |  | |  | |  |  |  |
| Right middle cingulum | 0 | -19 | 32 | 3 | 5.64 | | 0.001 | 1.000 | Nil |  |  | |  | |  |  |  |
| Unlabeled | -18 | 38 | 5 | 1 | 5.52 | | 0.001 | 1.000 | Nil |  |  | |  | |  |  |  |
| Left inferior parietal gyrus | -39 | -58 | 56 | 1 | 5.30 | | 0.001 | 1.000 | Nil |  |  | |  | |  |  |  |
| Right middle temporal gyrus | 66 | -40 | -13 | 1 | 5.28 | | 0.001 | 1.000 | Nil |  |  | |  | |  |  |  |
| Left middle temporal gyrus | Nil |  |  |  |  | |  |  | -60 | -64 | 2 | | 5 | | 13.17 | < 0.001 | 0.993 |
| Right inferior temporal gyrus | Nil |  |  |  |  | |  |  | 69 | -40 | -10 | | 7 | | 12.31 | < 0.001 | 0.997 |
| Right middle frontal gyrus | Nil |  |  |  |  | |  |  | 45 | 14 | 50 | | 2 | | 10.03 | < 0.001 | 1.000 |
| Right inferior orbitofrontal gyrus | Nil |  |  |  |  | |  |  | 45 | 35 | -10 | | 5 | | 9.15 | < 0.001 | 1.000 |
| Right inferior temporal gyrus | Nil |  |  |  |  | |  |  | 49 | -19 | -16 | | 9 | | 8.91 | < 0.001 | 1.000 |
| Left precentral gyrus | Nil |  |  |  |  | |  |  | -54 | 5 | 38 | | 1 | | 8.07 | < 0.001 | 1.000 |
| Right middle frontal gyrus | Nil |  |  |  |  | |  |  | 42 | 29 | 35 | | 2 | | 7.45 | < 0.001 | 1.000 |
| Right inferior orbitofrontal gyrus | Nil |  |  |  |  | |  |  | 57 | 35 | -7 | | 1 | | 7.45 | < 0.001 | 1.000 |
| Left precentral gyrus | Nil |  |  |  |  | |  |  | -48 | 5 | 47 | | 2 | | 7.38 | < 0.001 | 1.000 |
| Unlabeled | Nil |  |  |  |  | |  |  | -9 | 14 | 20 | | 1 | | 7.31 | < 0.001 | 1.000 |
| Left superior frontal gyrus | Nil |  |  |  |  | |  |  | -21 | 8 | 65 | | 1 | | 7.26 | < 0.001 | 1.000 |
| Right fusiform gyrus | Nil |  |  |  |  | |  |  | 42 | -34 | -10 | | 2 | | 7.16 | < 0.001 | 1.000 |
| Left thalamus | Nil |  |  |  |  | |  |  | -15 | -4 | 5 | | 1 | | 6.51 | 0.001 | 1.000 |
| Right inferior temporal gyrus | Nil |  |  |  |  | |  |  | 63 | -58 | -4 | | 1 | | 6.45 | 0.001 | 1.000 |
| Right middle cingulum | Nil |  |  |  |  | |  |  | 15 | -16 | 35 | | 1 | | 6.35 | 0.001 | 1.000 |
| Left superior temporal gyrus | Nil |  |  |  |  | |  |  | -63 | -7 | 8 | | 1 | | 6.34 | 0.001 | 1.000 |
| Right inferior temporal gyrus | Nil |  |  |  |  | |  |  | 45 | -10 | -25 | | 1 | | 6.20 | 0.001 | 1.000 |
| Right inferior temporal gyrus | Nil |  |  |  |  | |  |  | 60 | -61 | -7 | | 2 | | 6.19 | 0.001 | 1.000 |
| Left middle occipital gyrus | Nil |  |  |  |  | |  |  | -30 | -97 | -4 | | 1 | | 6.06 | 0.001 | 1.000 |
| Left thalamus | Nil |  |  |  |  | |  |  | -3 | -13 | 5 | | 1 | | 6.05 | 0.001 | 1.000 |
| Right operculum of the inferior frontal gyrus | Nil |  |  |  |  | |  |  | 48 | 11 | 11 | | 1 | | 6.04 | 0.001 | 1.000 |
| Left inferior occipital gyrus | Nil |  |  |  |  | |  |  | -33 | -94 | -13 | | 1 | | 5.96 | 0.001 | 1.000 |
|  | | | | | | | | | | | | | | | | | |
| **General illness awareness > Control stimuli** | | | | | | | | | | | | | | | | | |
| **Impaired** | | | | | | | | | | | | | | | | | |
| Left operculum of the rolandic gyrus | -39 | -19 | 17 | 6 | 8.93 | | < 0.001 | 0.989 | -39 | -19 | 17 | | 1 | | 6.19 | 0.001 | 1.000 |
| Right hippocampus | 30 | -10 | -13 | 3 | 8.01 | | < 0.001 | 0.998 | Nil |  |  | |  | |  |  |  |
| Left superior temporal gyrus | -51 | -31 | 17 | 1 | 6.02 | | < 0.001 | 1.000 | Nil |  |  | |  | |  |  |  |
| Right operculum of the rolandic gyrus | 42 | -13 | 20 | 1 | 5.36 | | 0.001 | 1.000 | Nil |  |  | |  | |  |  |  |
| Unlabeled | Nil |  |  |  |  | |  |  | 36 | -28 | -1 | | 1 | | 7.09 | < 0.001 | 1.000 |
|  | | | | | | | | | | | | | | | | | |
| **Intact** | | | | | | | | | | | | | | | | | |
| Left inferior orbital frontal gyrus | -45 | 44 | -13 | 4 | 7.18 | | < 0.001 | 1.000 | Nil |  |  | |  | |  |  |  |
| Left inferior temporal gyrus | -60 | -55 | -10 | 4 | 6.65 | | < 0.001 | 1.000 | Nil |  |  | |  | |  |  |  |
| Right middle frontal gyrus | 45 | 14 | 50 | 2 | 6.39 | | < 0.001 | 1.000 | 45 | 14 | 50 | | 2 | | 8.31 | < 0.001 | 1.000 |
| Right angular gyrus | 42 | -58 | 53 | 4 | 6.22 | | < 0.001 | 1.000 | Nil |  |  | |  | |  |  |  |
| Left middle occipital gyrus | Nil |  |  |  |  | |  |  | -30 | -97 | -4 | | 7 | | 17.54 | < 0.001 | 0.284 |
| Right inferior orbitofrontal gyrus | Nil |  |  |  |  | |  |  | 48 | 32 | -7 | | 3 | | 15.57 | < 0.001 | 0.510 |
| Right middle temporal gyrus | Nil |  |  |  |  | |  |  | 48 | -7 | -22 | | 1 | | 11.49 | < 0.001 | 0.999 |
| Left inferior frontal gyrus (triangular part) | Nil |  |  |  |  | |  |  | -48 | 35 | 14 | | 5 | | 8.70 | < 0.001 | 1.000 |
| Right inferior temporal gyrus | Nil |  |  |  |  | |  |  | 45 | -7 | -28 | | 1 | | 8.33 | < 0.001 | 1.000 |
| Right middle frontal gyrus | Nil |  |  |  |  | |  |  | 39 | 14 | 41 | | 3 | | 7.86 | < 0.001 | 1.000 |
| Right middle cingulum | Nil |  |  |  |  | |  |  | 12 | 32 | 35 | | 2 | | 7.75 | < 0.001 | 1.000 |
| Right middle cingulum | Nil |  |  |  |  | |  |  | 9 | 44 | 32 | | 7 | | 7.42 | < 0.001 | 1.000 |
| Unlabeled | Nil |  |  |  |  | |  |  | -27 | -25 | 17 | | 1 | | 6.54 | 0.001 | 1.000 |
| Right inferior temporal gyrus | Nil |  |  |  |  | |  |  | 66 | -43 | -13 | | 1 | | 6.23 | 0.001 | 1.000 |
| Right middle temporal gyrus | Nil |  |  |  |  | |  |  | 45 | -31 | -7 | | 1 | | 6.17 | 0.001 | 1.000 |
| Right anterior cingulum | Nil |  |  |  |  | |  |  | 12 | 38 | 23 | | 1 | | 6.06 | 0.001 | 1.000 |
| Right inferior orbitofrontal gyrus | Nil |  |  |  |  | |  |  | 57 | 35 | -7 | | 1 | | 6.03 | 0.001 | 1.000 |
|  | | | | | | | | | | | | | | | | | |
| **Symptom awareness > Control stimuli** | | | | | | | | | | | | | | | | | |
| **Impaired** | | | | | | | | | | | | | | | | | |
| Brain stem | 12 | -31 | -34 | 1 | 5.28 | | 0.001 | 1.000 | Nil |  |  | |  | |  |  |  |
| Right cerebellum crus VIII | Nil |  |  |  |  | |  |  | 21 | -52 | -46 | | 1 | | 9.82 | < 0.001 | 1.000 |
| Right cerebellum crus VIII | Nil |  |  |  |  | |  |  | 24 | -49 | -43 | | 1 | | 6.94 | < 0.001 | 1.000 |
|  | | | | | | | | | | | | | | | | | |
| **Intact** | | | | | | | | | | | | | | | | | |
| Left operculum of the frontal gyrus | -51 | 17 | 35 | 2 | 7.66 | | < 0.001 | 0.996 | Nil |  |  | |  | |  |  |  |
| Right fusiform gyrus | 30 | -70 | -1 | 1 | 7.65 | | < 0.001 | 0.996 | Nil |  |  | |  | |  |  |  |
| Left precentral gyrus | -48 | 8 | 47 | 1 | 6.58 | | < 0.001 | 1.000 | -48 | 8 | 47 | | 4 | | 7.30 | < 0.001 | 1.000 |
| Brain stem | 3 | -7 | -7 | 1 | 6.44 | | < 0.001 | 1.000 | Nil |  |  | |  | |  |  |  |
| Right putamen | 21 | 23 | -7 | 1 | 6.21 | | < 0.001 | 1.000 | Nil |  |  | |  | |  |  |  |
| Right superior orbital frontal gyrus | 18 | 23 | -16 | 1 | 5.96 | | < 0.001 | 1.000 | Nil |  |  | |  | |  |  |  |
| Left middle temporal gyrus | -66 | -52 | -4 | 2 | 5.84 | | 0.001 | 1.000 | Nil |  |  | |  | |  |  |  |
| Left caudate nucleus | -9 | 11 | 20 | 1 | 5.77 | | 0.001 | 1.000 | -9 | 11 | 20 | | 1 | | 6.10 | 0.001 | 1.000 |
| Left middle frontal gyrus | -27 | 29 | 50 | 1 | 5.65 | | 0.001 | 1.000 | Nil |  |  | |  | |  |  |  |
| Right postcentral gyrus | 63 | -4 | 29 | 1 | 5.45 | | 0.001 | 1.000 | Nil |  |  | |  | |  |  |  |
| Right postcentral gyrus | 66 | -7 | 26 | 1 | 5.23 | | 0.001 | 1.000 | Nil |  |  | |  | |  |  |  |
| Left precentral gyrus | Nil |  |  |  |  | |  |  | -51 | 2 | 38 | | 6 | | 9.68 | < 0.001 | 1.000 |
| Right fusiform gyrus | Nil |  |  |  |  | |  |  | 39 | -34 | -10 | | 4 | | 9.54 | < 0.001 | 1.000 |
| Right middle cingulum | Nil |  |  |  |  | |  |  | 15 | -1 | 38 | | 1 | | 8.95 | < 0.001 | 1.000 |
| Right middle temporal gyrus | Nil |  |  |  |  | |  |  | 69 | -37 | -10 | | 3 | | 8.52 | < 0.001 | 1.000 |
| Left superior temporal gyrus | Nil |  |  |  |  | |  |  | -42 | -19 | -1 | | 1 | | 8.45 | < 0.001 | 1.000 |
| Left insula | Nil |  |  |  |  | |  |  | -45 | -4 | -4 | | 11 | | 8.16 | < 0.001 | 1.000 |
| Right fusiform gyrus | Nil |  |  |  |  | |  |  | 33 | -55 | -10 | | 1 | | 7.61 | < 0.001 | 1.000 |
| Right middle temporal gyrus | Nil |  |  |  |  | |  |  | 60 | -64 | -1 | | 4 | | 7.56 | < 0.001 | 1.000 |
| Left superior frontal gyrus | Nil |  |  |  |  | |  |  | -18 | 8 | 65 | | 2 | | 7.46 | < 0.001 | 1.000 |
| Left inferior frontal gyrus (triangular part) | Nil |  |  |  |  | |  |  | -54 | 17 | 32 | | 1 | | 7.34 | < 0.001 | 1.000 |
| Left postcentral gyrus | Nil |  |  |  |  | |  |  | -66 | -16 | 23 | | 2 | | 7.31 | < 0.001 | 1.000 |
| Left inferior temporal gyrus | Nil |  |  |  |  | |  |  | -48 | -43 | -13 | | 3 | | 7.31 | < 0.001 | 1.000 |
| Right inferior orbitofrontal gyrus | Nil |  |  |  |  | |  |  | 54 | 41 | -13 | | 2 | | 7.29 | < 0.001 | 1.000 |
| Right precuneus | Nil |  |  |  |  | |  |  | 6 | -43 | 56 | | 1 | | 7.09 | < 0.001 | 1.000 |
| Right precuneus | Nil |  |  |  |  | |  |  | 9 | -40 | 53 | | 2 | | 6.89 | < 0.001 | 1.000 |
| Left supplementary motor area | Nil |  |  |  |  | |  |  | -6 | 2 | 53 | | 2 | | 6.86 | 0.001 | 1.000 |
| Right fusiform gyrus | Nil |  |  |  |  | |  |  | 39 | -19 | -22 | | 1 | | 6.86 | 0.001 | 1.000 |
| Right fusiform gyrus | Nil |  |  |  |  | |  |  | 45 | -19 | -16 | | 1 | | 6.86 | 0.001 | 1.000 |
| Right middle occipital gyrus | Nil |  |  |  |  | |  |  | 30 | -91 | 2 | | 1 | | 6.85 | 0.001 | 1.000 |
| Right fusiform gyrus | Nil |  |  |  |  | |  |  | 39 | -31 | -19 | | 3 | | 6.83 | 0.001 | 1.000 |
| Right precentral gyrus | Nil |  |  |  |  | |  |  | 42 | -13 | 38 | | 1 | | 6.76 | 0.001 | 1.000 |
| Unlabeled | Nil |  |  |  |  | |  |  | 15 | -16 | 32 | | 1 | | 6.66 | 0.001 | 1.000 |
| Right middle temporal gyrus | Nil |  |  |  |  | |  |  | 63 | -58 | -1 | | 1 | | 6.61 | 0.001 | 1.000 |
| Right operculum of the Rolandic gyrus | Nil |  |  |  |  | |  |  | 66 | -1 | 11 | | 2 | | 6.53 | 0.001 | 1.000 |
| Left middle frontal gyrus | Nil |  |  |  |  | |  |  | -30 | 56 | 11 | | 1 | | 6.30 | 0.001 | 1.000 |
| Unlabeled | Nil |  |  |  |  | |  |  | 21 | -19 | 26 | | 2 | | 6.24 | 0.001 | 1.000 |
| Unlabeled | Nil |  |  |  |  | |  |  | 0 | -10 | -4 | | 1 | | 6.12 | 0.001 | 1.000 |
| Right inferior temporal gyrus | Nil |  |  |  |  | |  |  | 42 | -52 | -7 | | 1 | | 6.10 | 0.001 | 1.000 |
| Right fusiform gyrus | Nil |  |  |  |  | |  |  | 39 | -40 | -19 | | 1 | | 6.06 | 0.001 | 1.000 |
| Left middle temporal gyrus | Nil |  |  |  |  | |  |  | -57 | -67 | 2 | | 1 | | 5.95 | 0.001 | 1.000 |
| Right middle temporal gyrus | Nil |  |  |  |  | |  |  | 69 | -43 | -7 | | 1 | | 5.90 | 0.001 | 1.000 |
|  | | | | | | | | | | | | | | | | | |
| **Need for treatment > Control stimuli** | | | | | | | | | | | | | | | | | |
| **Impaired** | | | | | | | | | | | | | | | | | |
| Left middle frontal gyrus | -30 | 44 | 29 | 1 | 5.46 | | 0.001 | 1.000 | Nil |  |  | |  | |  |  |  |
|  | | | | | | | | | | | | | | | | | |
| **Intact** | | | | | | | | | | | | | | | | | |
| Right middle cingulum | 0 | -19 | 32 | 12 | 9.05 | | < 0.001 | 0.978 | 0 | -19 | 32 | | 14 | | 19.46 | < 0.001 | 0.170 |
| Right medial orbital frontal gyrus | 6 | 53 | -1 | 12 | 8.65 | | < 0.001 | 0.987 | Nil |  |  | |  | |  |  |  |
| Right anterior cingulum | 6 | 32 | 2 | 4 | 8.62 | | < 0.001 | 0.987 | Nil |  |  | |  | |  |  |  |
| Right lingual gyrus | 6 | -37 | -1 | 3 | 8.13 | | < 0.001 | 0.994 | 6 | -37 | -1 | | 1 | | 6.21 | 0.001 | 1.000 |
| Left supplementary motor area | -9 | 20 | 62 | 1 | 7.60 | | < 0.001 | 0.998 | Nil |  |  | |  | |  |  |  |
| Left superior temporal gyrus | -42 | -40 | 20 | 1 | 7.51 | | < 0.001 | 0.998 | -42 | -40 | 20 | | 1 | | 5.92 | 0.001 | 1.000 |
| Left inferior orbital frontal gyrus | -33 | 44 | -19 | 1 | 6.33 | | < 0.001 | 1.000 | -33 | 44 | -19 | | 1 | | 6.64 | 0.001 | 1.000 |
| Left middle frontal gyrus | -33 | 5 | 59 | 2 | 6.27 | | < 0.001 | 1.000 | Nil |  |  | |  | |  |  |  |
| Left superior frontal gyrus | -51 | -13 | 2 | 1 | 6.24 | | < 0.001 | 1.000 | Nil |  |  | |  | |  |  |  |
| Left inferior temporal gyrus | -60 | -61 | -7 | 3 | 6.22 | | < 0.001 | 1.000 | Nil |  |  | |  | |  |  |  |
| Right medial orbital frontal gyrus | 0 | 47 | -4 | 1 | 6.15 | | < 0.001 | 1.000 | 0 | 47 | -4 | | 1 | | 6.91 | < 0.001 | 1.000 |
| Left angular gyrus | -57 | -64 | 32 | 3 | 5.66 | | 0.001 | 1.000 | Nil |  |  | |  | |  |  |  |
| Unlabeled | -18 | 47 | -1 | 1 | 5.55 | | 0.001 | 1.000 | Nil |  |  | |  | |  |  |  |
| Unlabeled | 3 | 14 | 20 | 1 | 5.45 | | 0.001 | 1.000 | Nil |  |  | |  | |  |  |  |
| Left middle temporal gyrus | Nil |  |  |  |  | |  |  | -60 | -64 | 5 | | 5 | | 43.54 | < 0.001 | 0.003* |
| Right middle cingulum | Nil |  |  |  |  | |  |  | 12 | -28 | 44 | | 1 | | 19.60 | < 0.001 | 0.164 |
| Left thalamus | Nil |  |  |  |  | |  |  | -3 | -13 | 5 | | 3 | | 15.74 | < 0.001 | 0.484 |
| Right superior temporal gyrus | Nil |  |  |  |  | |  |  | 63 | -52 | 23 | | 2 | | 14.46 | < 0.001 | 0.734 |
| Right middle temporal gyrus | Nil |  |  |  |  | |  |  | 60 | -25 | -10 | | 16 | | 14.21 | < 0.001 | 0.801 |
| Unlabeled | Nil |  |  |  |  | |  |  | -6 | 17 | 20 | | 65 | | 13.19 | < 0.001 | 0.994 |
| Right olfactory bulb | Nil |  |  |  |  | |  |  | 21 | 14 | -16 | | 2 | | 12.07 | < 0.001 | 0.998 |
| Left operculum of the Rolandic gyrus | Nil |  |  |  |  | |  |  | -63 | -7 | 11 | | 1 | | 12.06 | < 0.001 | 0.998 |
| Unlabeled | Nil |  |  |  |  | |  |  | 9 | 26 | -4 | | 18 | | 11.57 | < 0.001 | 0.999 |
| Right operculum of the Rolandic gyrus | Nil |  |  |  |  | |  |  | 51 | 8 | 11 | | 2 | | 8.98 | < 0.001 | 1.000 |
| Left amygdala | Nil |  |  |  |  | |  |  | -24 | 2 | -22 | | 4 | | 8.86 | < 0.001 | 1.000 |
| Right putamen | Nil |  |  |  |  | |  |  | 36 | -1 | 2 | | 1 | | 8.42 | < 0.001 | 1.000 |
| Left cerebellum crus IV and V | Nil |  |  |  |  | |  |  | -9 | -40 | -10 | | 3 | | 7.87 | < 0.001 | 1.000 |
| Unlabeled | Nil |  |  |  |  | |  |  | 9 | 2 | -7 | | 1 | | 7.86 | < 0.001 | 1.000 |
| Left precentral gyrus | Nil |  |  |  |  | |  |  | -48 | 8 | 47 | | 1 | | 7.74 | < 0.001 | 1.000 |
| Right superior medial frontal gyrus | Nil |  |  |  |  | |  |  | 9 | 50 | 2 | | 6 | | 7.65 | < 0.001 | 1.000 |
| Right supramarginal gyrus | Nil |  |  |  |  | |  |  | 60 | -43 | 44 | | 1 | | 7.56 | < 0.001 | 1.000 |
| Left inferior parietal gyrus | Nil |  |  |  |  | |  |  | -60 | -43 | 41 | | 2 | | 7.52 | < 0.001 | 1.000 |
| Right middle temporal gyrus | Nil |  |  |  |  | |  |  | 51 | -40 | 11 | | 4 | | 7.45 | < 0.001 | 1.000 |
| Left superior temporal gyrus | Nil |  |  |  |  | |  |  | -66 | -25 | 11 | | 8 | | 7.36 | < 0.001 | 1.000 |
| Right parahippocampal area | Nil |  |  |  |  | |  |  | 18 | -19 | -22 | | 1 | | 7.27 | < 0.001 | 1.000 |
| Unlabeled | Nil |  |  |  |  | |  |  | 12 | -25 | -25 | | 1 | | 7.15 | < 0.001 | 1.000 |
| Right insula | Nil |  |  |  |  | |  |  | 39 | 23 | -4 | | 1 | | 7.12 | < 0.001 | 1.000 |
| Left middle cingulum | Nil |  |  |  |  | |  |  | -12 | 5 | 35 | | 1 | | 7.11 | < 0.001 | 1.000 |
| Left superior frontal gyrus | Nil |  |  |  |  | |  |  | -18 | 5 | 65 | | 5 | | 7.11 | < 0.001 | 1.000 |
| Right middle temporal gyrus | Nil |  |  |  |  | |  |  | 48 | -16 | -13 | | 4 | | 7.02 | < 0.001 | 1.000 |
| Right inferior temporal gyrus | Nil |  |  |  |  | |  |  | 69 | -40 | -10 | | 1 | | 6.98 | < 0.001 | 1.000 |
| Unlabeled | Nil |  |  |  |  | |  |  | -18 | 35 | -4 | | 1 | | 6.96 | < 0.001 | 1.000 |
| Left superior temporal gyrus | Nil |  |  |  |  | |  |  | -45 | 5 | -10 | | 1 | | 6.96 | < 0.001 | 1.000 |
| Right middle temporal gyrus | Nil |  |  |  |  | |  |  | 60 | -37 | 8 | | 1 | | 6.57 | 0.001 | 1.000 |
| Right insula | Nil |  |  |  |  | |  |  | 36 | 14 | 14 | | 1 | | 6.44 | 0.001 | 1.000 |
| Left middle temporal gyrus | Nil |  |  |  |  | |  |  | -57 | -70 | 8 | | 2 | | 6.38 | 0.001 | 1.000 |
| Right superior temporal gyrus | Nil |  |  |  |  | |  |  | 66 | -4 | 8 | | 2 | | 6.30 | 0.001 | 1.000 |
| Right angular gyrus | Nil |  |  |  |  | |  |  | 42 | -55 | 56 | | 2 | | 6.28 | 0.001 | 1.000 |
| Left inferior frontal gyrus (triangular part) | Nil |  |  |  |  | |  |  | -48 | 38 | 14 | | 1 | | 6.22 | 0.001 | 1.000 |
| Right putamen | Nil |  |  |  |  | |  |  | 33 | 11 | -1 | | 1 | | 6.22 | 0.001 | 1.000 |
| Left superior medial frontal gyrus | Nil |  |  |  |  | |  |  | -6 | 23 | 44 | | 1 | | 6.18 | 0.001 | 1.000 |
| Right inferior parietal lobule | Nil |  |  |  |  | |  |  | 48 | -43 | 56 | | 1 | | 6.17 | 0.001 | 1.000 |
| Right inferior temporal gyrus | Nil |  |  |  |  | |  |  | 63 | -58 | -4 | | 1 | | 6.11 | 0.001 | 1.000 |
| Right cerebellum crus IV and V | Nil |  |  |  |  | |  |  | 12 | -37 | -10 | | 2 | | 6.09 | 0.001 | 1.000 |
| Left superior parietal lobule | Nil |  |  |  |  | |  |  | -30 | -70 | 53 | | 1 | | 6.01 | 0.001 | 1.000 |
| Right fusiform gyrus | Nil |  |  |  |  | |  |  | 42 | -37 | -13 | | 1 | | 6.00 | 0.001 | 1.000 |
| Left operculum of the Rolandic gyrus | Nil |  |  |  |  | |  |  | -48 | 5 | 11 | | 1 | | 5.98 | 0.001 | 1.000 |
| Left inferior frontal gyrus (triangular part) | Nil |  |  |  |  | |  |  | -42 | 44 | 14 | | 1 | | 5.92 | 0.001 | 1.000 |
| Right superior orbitofrontal gyrus | Nil |  |  |  |  | |  |  | 18 | 31 | -16 | | 1 | | 5.90 | 0.001 | 1.000 |
|  | | | | | | | | | | | | | | | | | |
| ***SUD subgroup only*** | | | | | | | | | | | | | | | | | |
| **Illness-related > Control stimuli** | | | | | | | | | | | | | | | | | |
| **Impaired** | | | | | | | | | | | | | | | | | |
| Left caudate | -6 | 2 | 17 | 1 | 7.76 | | < 0.001 | 0.995 | Nil |  |  | |  | |  |  |  |
| Left putamen | -30 | -1 | 11 | 1 | 6.57 | | < 0.001 | 1.000 | Nil |  |  | |  | |  |  |  |
| Right middle frontal gyrus | 39 | 11 | 56 | 1 | 6.32 | | < 0.001 | 1.000 | Nil |  |  | |  | |  |  |  |
| Unlabelled | -21 | -43 | 17 | 2 | 6.07 | | < 0.001 | 1.000 | Nil |  |  | |  | |  |  |  |
| Left caudate | -9 | 26 | -4 | 1 | 5.99 | | < 0.001 | 1.000 | Nil |  |  | |  | |  |  |  |
| Unlabelled | -18 | -10 | 32 | 1 | 5.56 | | 0.001 | 1.000 | Nil |  |  | |  | |  |  |  |
| Cerebellar vermis | -3 | -46 | -37 | 2 | 5.49 | | 0.001 | 1.000 | Nil |  |  | |  | |  |  |  |
| Left cerebellum crus IX | -9 | -1 | 20 | 1 | 5.47 | | 0.001 | 1.000 | Nil |  |  | |  | |  |  |  |
| Unlabeled | Nil |  |  |  |  | |  |  | 3 | 2 | 14 | | 3 | | 12.06 | < 0.001 | 0.992 |
| Right inferior temporal gyrus | Nil |  |  |  |  | |  |  | 45 | -55 | -10 | | 1 | | 6.65 | 0.001 | 1.000 |
| Left middle cingulum | Nil |  |  |  |  | |  |  | -15 | -1 | 44 | | 1 | | 6.64 | 0.001 | 1.000 |
|  | | | | | | | | | | | | | | | | | |
| **Intact** | | | | | | | | | | | | | | | | | |
| Left middle occipital gyrus | Nil |  |  |  |  |  | |  | -42 | -73 | 14 | | 1 | | 9.05 | < 0.001 | 1.000 |
| Left middle frontal gyrus | Nil |  |  |  |  |  | |  | -39 | 41 | 29 | | 1 | | 7.46 | < 0.001 | 1.000 |
| Unlabeled | Nil |  |  |  |  |  | |  | 15 | -22 | -16 | | 1 | | 7.19 | < 0.001 | 1.000 |
| Left supramarginal gyrus | Nil |  |  |  |  |  | |  | -63 | -34 | 41 | | 1 | | 6.33 | 0.001 | 1.000 |
| Left precuneus | Nil |  |  |  |  |  | |  | -3 | -40 | 62 | | 1 | | 5.99 | 0.001 | 1.000 |
| Left insula | Nil |  |  |  |  |  | |  | -42 | -1 | 2 | | 1 | | 5.97 | 0.001 | 1.000 |
|  | | | | | | | | | | | | | | | | | |
| **General illness awareness > Control stimuli** | | | | | | | | | | | | | | | | | |
| **Impaired** | | | | | | | | | | | | | | | | | |
| Unlabelled | -12 | -34 | -31 | 1 | 7.29 | | < 0.001 | 1.000 | Nil |  |  | |  | |  |  |  |
| Unlabelled | -15 | -37 | -34 | 1 | 7.07 | | < 0.001 | 1.000 | Nil |  |  | |  | |  |  |  |
| Right superior frontal gyrus | 24 | 59 | 14 | 2 | 6.51 | | < 0.001 | 1.000 | Nil |  |  | |  | |  |  |  |
| Right middle frontal gyrus | 39 | 11 | 56 | 1 | 6.25 | | < 0.001 | 1.000 | Nil |  |  | |  | |  |  |  |
| Right middle frontal gyrus | 33 | 2 | 62 | 1 | 6.03 | | < 0.001 | 1.000 | Nil |  |  | |  | |  |  |  |
| Right superior frontal gyrus | 18 | 32 | 41 | 2 | 6.03 | | < 0.001 | 1.000 | 18 | 32 | 41 | | 1 | | 7.19 | < 0.001 | 1.000 |
| Cerebellar vermis | -3 | -37 | -34 | 1 | 5.72 | | 0.001 | 1.000 | Nil |  |  | |  | |  |  |  |
| Left cerebellum crus IX | Nil |  |  |  |  | |  |  | -6 | -46 | -34 | | 6 | | 12.46 | < 0.001 | 0.995 |
| Left cerebellum crus II | Nil |  |  |  |  | |  |  | -21 | -76 | -43 | | 1 | | 6.18 | 0.001 | 1.000 |
| Unlabeled | Nil |  |  |  |  | |  |  | 0 | -37 | -28 | | 1 | | 6.12 | 0.001 | 1.000 |
| Left middle cingulum | Nil |  |  |  |  | |  |  | -12 | 2 | 41 | | 1 | | 6.10 | 0.001 | 1.000 |
| Right precentral gyrus | Nil |  |  |  |  | |  |  | 36 | -4 | 35 | | 1 | | 6.03 | 0.001 | 1.000 |
|  | | | | | | | | | | | | | | | | | |
| **Intact** | | | | | | | | | | | | | | | | | |
| Right superior temporal gyrus | 48 | -7 | -1 | 1 | 6.14 | | < 0.001 | 1.000 | Nil |  |  | |  | |  |  |  |
| Left inferior parietal lobule | Nil |  |  |  |  | |  |  | -60 | -37 | 44 | | 1 | | 7.16 | < 0.001 | 1.000 |
| Left precuneus | Nil |  |  |  |  | |  |  | -3 | -46 | 68 | | 3 | | 6.76 | 0.001 | 1.000 |
| Right paracentral lobule | Nil |  |  |  |  | |  |  | 0 | -40 | 65 | | 1 | | 6.03 | 0.001 | 1.000 |
|  | | | | | | | | | | | | | | | | | |
| **Symptom awareness > Control stimuli** | | | | | | | | | | | | | | | | | |
| **Impaired** | | | | | | | | | | | | | | | | | |
| Right cerebellum crus IX | 3 | -46 | -37 | 4 | 7.01 | | < 0.001 | 0.999 | Nil |  |  | |  | |  |  |  |
| Unlabeled | 27 | -58 | 20 | 2 | 6.67 | | < 0.001 | 1.000 | Nil |  |  | |  | |  |  |  |
| Right caudate | 9 | 2 | 17 | 1 | 5.96 | | < 0.001 | 1.000 | Nil |  |  | |  | |  |  |  |
| Left putamen | -24 | -13 | 14 | 1 | 5.55 | | 0.001 | 1.000 | Nil |  |  | |  | |  |  |  |
| Unlabeled | Nil |  |  |  |  | |  |  | 6 | -1 | 17 | | 1 | | 6.10 | 0.001 | 1.000 |
| Unlabeled | Nil |  |  |  |  | |  |  | 27 | -40 | 29 | | 1 | | 6.03 | 0.001 | 1.000 |
|  | | | | | | | | | | | | | | | | | |
| **Intact** | | | | | | | | | | | | | | | | | |
| Left operculum of the Rolandic gyrus | Nil |  |  |  |  | |  |  | -42 | -22 | 23 | | 1 | | 8.00 | < 0.001 | 1.000 |
| Left insula | Nil |  |  |  |  | |  |  | -36 | -4 | 8 | | 2 | | 7.23 | < 0.001 | 1.000 |
| Left thalamus | Nil |  |  |  |  | |  |  | -24 | -22 | 14 | | 2 | | 7.16 | < 0.001 | 1.000 |
| Left middle frontal gyrus | Nil |  |  |  |  | |  |  | -42 | 35 | 29 | | 3 | | 6.74 | 0.001 | 1.000 |
| Left thalamus | Nil |  |  |  |  | |  |  | -18 | -16 | 8 | | 2 | | 6.41 | 0.001 | 1.000 |
| Left middle occipital gyrus | Nil |  |  |  |  | |  |  | -42 | -82 | 26 | | 1 | | 6.04 | 0.001 | 1.000 |
| Right precuneus | Nil |  |  |  |  | |  |  | 12 | -49 | 20 | | 1 | | 6.01 | 0.001 | 1.000 |
|  | | | | | | | | | | | | | | | | | |
| **Need for treatment > Control stimuli** | | | | | | | | | | | | | | | | | |
| **Impaired** | | | | | | | | | | | | | | | | | |
| Unlabeled | -6 | -1 | 20 | 2 | 5.38 | | 0.001 | 1.000 | Nil |  |  | |  | |  |  |  |
| Left middle cingulum | -15 | -10 | 38 | 1 | 5.29 | | 0.001 | 1.000 | -15 | -10 | 38 | | 1 | | 6.36 | 0.001 | 1.000 |
| Unlabeled | -18 | -40 | 20 | 1 | 5.29 | | 0.001 | 1.000 | Nil |  |  | |  | |  |  |  |
| Left superior frontal gyrus | -24 | 2 | 65 | 1 | 5.23 | | 0.001 | 1.000 | Nil |  |  | |  | |  |  |  |
| Unlabeled | -21 | -43 | 17 | 1 | 5.22 | | 0.001 | 1.000 | Nil |  |  | |  | |  |  |  |
|  | | | | | | | | | | | | | | | | | |
| **Intact** | | | | | | | | | | | | | | | | | |
| Right cerebellum crus VIII | Nil |  |  |  |  | |  |  | 21 | -46 | -40 | | 2 | | 13.09 | < 0.001 | 0.987 |
| Left middle frontal gyrus | Nil |  |  |  |  | |  |  | -39 | 41 | 29 | | 4 | | 11.10 | < 0.001 | 0.997 |
| Left calcarine sulcus | Nil |  |  |  |  | |  |  | -9 | -55 | 8 | | 12 | | 9.76 | < 0.001 | 1.000 |
| Right parahippocampal area | Nil |  |  |  |  | |  |  | 15 | -22 | -13 | | 7 | | 8.18 | < 0.001 | 1.000 |
| Right posterior cingulum | Nil |  |  |  |  | |  |  | 6 | -43 | 14 | | 2 | | 6.41 | 0.001 | 1.000 |
| Left insula | Nil |  |  |  |  | |  |  | -42 | 2 | 2 | | 1 | | 6.13 | 0.001 | 1.000 |
| Note: Threshold: *p* < 0.001, uncorr., 0 voxels;  **p* < 0.05 FWE corrected | | | | | | | | | | | | | | | | | |

| **Supplementary Table 3. Regional activations for the second-level contrast for AUD and SUD combined – Group Comparison** | | | | | | | | | | | | | | | | | | | | | |
| --- | --- | --- | --- | --- | --- | --- | --- | --- | --- | --- | --- | --- | --- | --- | --- | --- | --- | --- | --- | --- | --- |
|  | **Adjusted for age and gender** | | | | | | | **Adjusted for age, gender, and substance category (i.e., alcohol and substance)** | | | | | | | **Adjusted for age, gender, substance category (i.e., alcohol and substance), and illness severity (i.e., AUDIT/DUDIT scores)** | | | | | | |
|  | Cluster Maxima | | | Cluster size | *t* value | *p* value uncorrected | *p* value FWE corrected | Cluster Maxima | | | Cluster size | *t* value | *p* value uncorrected | *p* value FWE corrected | Cluster Maxima | | | Cluster size | *t* value | *p* value uncorrected | *p* value FWE corrected |
|  | x | y | z |  |  |  |  | x | y | z |  |  |  |  | x | y | z |  |  |  |  |
| **Illness-related > Control stimuli** | | | | | | | | | | | | | | |  | | | | | | |
| **Impaired > Intact** | | | | | | | | | | | | | | |  | | | | | | |
| Right insula | 33 | -19 | 17 | 1 | 3.98 | 0.001 | 0.990 | 33 | -19 | 17 | 1 | 3.75 | 0.001 | 0.999 | Nil |  |  |  |  |  |  |
| Left middle occipital gyrus | Nil |  |  |  |  |  |  | -36 | -76 | -1 | 2 | 4.22 | < 0.001 | 0.978 | -36 | -76 | -1 | 1 | 3.84 | 0.001 | 0.999 |
|  | | | | | | | | | | | | | | | | | | | | | |
| **Intact > Impaired** | | | | | | | | | | | | | | | | | | | | | |
| Right cerebellum crus II | Nil |  |  |  |  |  |  | 39 | -64 | -46 | 1 | 3.98 | 0.001 | 0.995 | Nil |  |  |  |  |  |  |
| Left precuneus | Nil |  |  |  |  |  |  | Nil |  |  |  |  |  |  | -15 | -49 | 20 | 4 | 4.97 | < 0.001 | 0.810 |
| Right thalamus | Nil |  |  |  |  |  |  | Nil |  |  |  |  |  |  | 18 | -10 | 8 | 2 | 3.91 | 0.001 | 0.998 |
| Right operculum of the Rolandic gyrus | Nil |  |  |  |  |  |  | Nil |  |  |  |  |  |  | 51 | 8 | 8 | 1 | 3.79 | 0.001 | 0.999 |
|  | | | | | | | | | | | | | | | | | | | | | |
| **General illness awareness > Control stimuli** | | | | | | | | | | | | | | | | | | | | | |
| **Impaired > Intact** | | | | | | | | | | | | | | | | | | | | | |
| Unlabeled | -66 | 37 | 26 | 11 | 4.66 | < 0.001 | 0.844 | -66 | -37 | 26 | 11 | 4.52 | < 0.001 | 0.934 | Nil |  |  |  |  |  |  |
| Right insula | 30 | -19 | 17 | 2 | 4.49 | < 0.001 | 0.907 | 30 | -19 | 17 | 2 | 4.25 | < 0.001 | 0.979 | Nil |  |  |  |  |  |  |
| Right caudate | 15 | -22 | 23 | 3 | 4.05 | < 0.001 | 0.989 | 15 | -22 | 23 | 2 | 3.98 | 0.001 | 0.996 | Nil |  |  |  |  |  |  |
| Left lingual gyrus | -21 | -82 | -1 | 2 | 4.01 | 0.001 | 0.991 | -21 | -82 | -1 | 2 | 4.03 | 0.001 | 0.995 | Nil |  |  |  |  |  |  |
| Unlabeled | -27 | -4 | 35 | 2 | 3.89 | 0.001 | 0.996 | -27 | -4 | 35 | 1 | 3.79 | 0.001 | 0.999 | Nil |  |  |  |  |  |  |
| Left caudate | -6 | -4 | 20 | 1 | 3.77 | 0.001 | 0.999 |  |  |  |  |  |  |  | Nil |  |  |  |  |  |  |
| Left caudate | -6 | 2 | 17 | 1 | 3.72 | 0.001 | 0.999 | -6 | 2 | 17 | 3 | 4.17 | < 0.001 | 0.987 | -6 | 2 | 17 | 2 | 4.22 | < 0.001 | 0.990 |
| Left paracentral lobule | Nil |  |  |  |  |  |  | -3 | -19 | 56 | 1 | 3.84 | 0.001 | 0.999 | Nil |  |  |  |  |  |  |
| Right precentral gyrus | Nil |  |  |  |  |  |  | Nil |  |  |  |  |  |  | 18 | -19 | 71 | 3 | 4.07 | 0.001 | 0.996 |
|  | | | | | | | | | | | | | | | | | | | | | |
| **Intact > Impaired** | | | | | | | | | | | | | | | | | | | | | |
| Unlabeled | -9 | -7 | -7 | 1 | 3.79 | 0.001 | 0.998 | Nil |  |  |  |  |  |  | -9 | -7 | -7 | 2 | 4.91 | < 0.001 | 0.849 |
| Unlabeled | Nil |  |  |  |  |  |  | Nil |  |  |  |  |  |  | 21 | -10 | 8 | 1 | 3.92 | 0.001 | 0.999 |
|  | | | | | | | | | | | | | | | | | | | | | |
| **Symptom awareness > Control stimuli** | | | | | | | | | | | | | | | | | | | | | |
| **Impaired > Intact** | | | | | | | | | | | | | | | | | | | | | |
| Right cerebellum crus IX | 9 | -43 | -37 | 1 | 3.74 | 0.001 | 0.999 | Nil |  |  |  |  |  |  | Nil |  |  |  |  |  |  |
| Right angular gyrus | 39 | -46 | 26 | 1 | 3.71 | 0.001 | 0.999 | Nil |  |  |  |  |  |  | Nil |  |  |  |  |  |  |
|  | | | | | | | | | | | | | | | | | | | | | |
| **Intact > Impaired** | | | | | | | | | | | | | | | | | | | | | |
| Right caudate | 15 | 26 | 14 | 3 | 4.37 | < 0.001 | 0.937 | 15 | 26 | 14 | 3 | 4.36 | < 0.001 | 0.959 | Nil |  |  |  |  |  |  |
| Right cerebellum crus II |  |  |  |  |  |  |  | 39 | -64 | -46 | 1 | 3.74 | 0.001 | 0.999 | Nil |  |  |  |  |  |  |
| Right operculum of the Rolandic gyrus | Nil |  |  |  |  |  |  | Nil |  |  |  |  |  |  | 51 | 8 | 8 | 6 | 4.88 | < 0.001 | 0.834 |
| Right superior temporal gyrus | Nil |  |  |  |  |  |  | Nil |  |  |  |  |  |  | 39 | -40 | -16 | 1 | 4.24 | < 0.001 | 0.984 |
| Right middle frontal gyrus | Nil |  |  |  |  |  |  | Nil |  |  |  |  |  |  | 36 | 47 | 29 | 1 | 3.95 | 0.001 | 0.997 |
| Left precuneus | Nil |  |  |  |  |  |  | Nil |  |  |  |  |  |  | -15 | -49 | 20 | 2 | 3.94 | 0.001 | 0.998 |
| Left superior temporal gyrus | Nil |  |  |  |  |  |  | Nil |  |  |  |  |  |  | -45 | -7 | -4 | 1 | 3.86 | 0.001 | 0.999 |
|  | | | | | | | | | | | | | | | | | | | | | |
| **Need for treatment > Control stimuli** | | | | | | | | | | | | | | | | | | | | | |
| **Impaired > Intact** | | | | | | | | | | | | | | | | | | | | | |
| Left putamen | -27 | -10 | 14 | 7 | 4.67 | < 0.001 | 0.822 | -27 | -10 | 14 | 6 | 4.52 | < 0.001 | 0.923 |  |  |  |  |  |  |  |
| Left middle occipital gyrus | -36 | -73 | 2 | 7 | 4.49 | < 0.001 | 0.895 | -36 | -73 | 2 | 11 | 5.01 | < 0.001 | 0.730 | -36 | -73 | 2 | 6 | 4.78 | < 0.001 | 0.886 |
| Unlabeled | -15 | -19 | 29 | 1 | 3.75 | 0.001 | 0.998 | Nil |  |  |  |  |  |  | Nil |  |  |  |  |  |  |
| Right operculum of the rolandic gyrus | 36 | -19 | 17 | 1 | 3.73 | 0.001 | 0.999 | Nil |  |  |  |  |  |  | Nil |  |  |  |  |  |  |
| Left putamen | Nil |  |  |  |  |  |  | Nil |  |  |  |  |  |  | -30 | -7 | 14 | 3 | 4.71 | < 0.001 | 0.909 |
|  | | | | | | | | | | | | | | | | | | | | | |
| **Intact > Impaired** | | | | | | | | | | | | | | | | | | | | | |
| Right insula | Nil |  |  |  |  |  |  | Nil |  |  |  |  |  |  | 42 | 11 | 2 | 7 | 5.25 | < 0.001 | 0.696 |
| Right thalamus | Nil |  |  |  |  |  |  | Nil |  |  |  |  |  |  | 18 | -10 | 8 | 3 | 5.09 | < 0.001 | 0.766 |
| Right anterior cingulum | Nil |  |  |  |  |  |  | Nil |  |  |  |  |  |  | 9 | 29 | 29 | 10 | 5.01 | < 0.001 | 0.802 |
| Left precuneus | Nil |  |  |  |  |  |  | Nil |  |  |  |  |  |  | -15 | -52 | 20 | 1 | 4.78 | < 0.001 | 0.889 |
| Right anterior cingulum | Nil |  |  |  |  |  |  | Nil |  |  |  |  |  |  | 3 | 32 | 20 | 29 | 4.62 | < 0.001 | 0.931 |
| Left superior temporal gyrus | Nil |  |  |  |  |  |  | Nil |  |  |  |  |  |  | -45 | -10 | -4 | 2 | 4.27 | < 0.001 | 0.985 |
| Right cerebellum crus II | Nil |  |  |  |  |  |  | Nil |  |  |  |  |  |  | 39 | -64 | -46 | 1 | 3/91 | 0.001 | 0.999 |
| Right anterior cingulum | Nil |  |  |  |  |  |  | Nil |  |  |  |  |  |  | 6 | 41 | -1 | 1 | 3.82 | 0.001 | 0.999 |
| Note: Threshold: *p* < 0.001, uncorr., 0 voxels;  No suprathreshold peak in association with intact > impaired was identified  **p* < 0.05 | | | | | | | | | | | | | | | | | | | | | |

| **Supplementary Table 4. Regional activations for the second-level contrast for AUD only and SUD only groups – Group Comparison** | | | | | | | | | | | | | | | |
| --- | --- | --- | --- | --- | --- | --- | --- | --- | --- | --- | --- | --- | --- | --- | --- |
|  | **Adjusted for age and gender** | | | | | | | **Adjusted for age, gender, and illness severity (i.e., AUDIT/DUDIT scores)** | | | | | | | |
|  | Cluster Maxima | | | Cluster size | *t* value | *p* value uncorrected | *p* value FWE corrected | Cluster Maxima | | | Cluster size | | *t* value | *p* value uncorrected | *p* value FWE corrected |
|  | x | y | z |  |  |  |  | x | y | z |  |  |  |  |  |
| ***AUD subgroup only*** | | | | | | | | | | | | | | | |
| **Illness-related > Control stimuli** | | | | | | | | | | | | | | | |
| **Impaired > Intact** | | | | | | | | | | | | | | | |
| Left superior temporal gyrus | -54 | -34 | 17 | 4 | 8.66 | < 0.001 | 0.834 | -54 | -34 | 17 | 1 | 6.34 | | 0.001 | 1.000 |
| Left insula | -39 | -1 | 11 | 1 | 5.93 | 0.001 | 0.972 | Nil |  |  |  |  | |  |  |
| Right precentral gyrus | 36 | -19 | 65 | 1 | 5.27 | 0.001 | 0.972 | Nil |  |  |  |  | |  |  |
|  | | | | | | | | | | | | | | | |
| **Intact > Impaired** | | | | | | | | | | | | | | | |
| Right medial superior frontal gyrus | 12 | 50 | 8 | 3 | 8.73 | < 0.001 | 0.981 | 12 | 50 | 8 | 1 | 6.67 | | 0.001 | 1.000 |
| Unlabeled | -27 | -43 | 23 | 2 | 6.34 | < 0.001 | 1.000 | -27 | -43 | 23 | 1 | 6.03 | | 0.001 | 1.000 |
| Unlabeled | Nil |  |  |  |  |  |  | -9 | -10 | -7 | 1 | 7.49 | | < 0.001 | 1.000 |
|  | | | | | | | | | | | | | | | |
| **General illness awareness > Control stimuli** | | | | | | | | | | | | | | | |
| **Impaired > Intact** | | | | | | | | | | | | | | | |
| Left superior temporal gyrus | -51 | -37 | 11 | 53 | 12.77 | < 0.001 | 0.364 | -51 | -37 | 11 | 1 | 11.72 | | < 0.001 | 1.000 |
| Unlabeled | -27 | -19 | 35 | 1 | 8.36 | < 0.001 | 0.996 | -27 | -19 | 35 | 1 | 6.34 | | 0.001 | 1.000 |
| Unlabeled | -36 | -37 | 26 | 2 | 8.00 | < 0.001 | 0.998 | -36 | -37 | 26 | 2 | 8.12 | | < 0.001 | 1.000 |
| Left cuneus | 0 | -88 | 23 | 3 | 7.96 | < 0.001 | 0.998 | 0 | -88 | 23 | 3 | 6.27 | | 0.001 | 1.000 |
| Left superior temporal gyrus | -42 | -22 | 5 | 3 | 7.32 | < 0.001 | 1.000 | Nil |  |  |  |  | |  |  |
| Right insula | 36 | -7 | -7 | 1 | 5.81 | 0.001 | 1.000 | Nil |  |  |  |  | |  |  |
| Left superior temporal gyrus | -51 | -19 | 11 | 1 | 5.76 | 0.001 | 1.000 | Nil |  |  |  |  | |  |  |
| Right precentral gyrus | 36 | -22 | 62 | 3 | 5.41 | 0.001 | 1.000 | Nil |  |  |  |  | |  |  |
| Unlabeled | Nil |  |  |  |  |  |  | -21 | -10 | 35 | 1 | 11.72 | | < 0.001 | 0.998 |
| Right caudate | Nil |  |  |  |  |  |  | 21 | 26 | 17 | 2 | 9.81 | | < 0.001 | 1.000 |
| Left superior temporal gyrus | Nil |  |  |  |  |  |  | -66 | -40 | 20 | 13 | 9.24 | | < 0.001 | 1.000 |
| Unlabeled | Nil |  |  |  |  |  |  | 18 | -31 | -31 | 8 | 8.98 | | < 0.001 | 1.000 |
| Left inferior temporal gyrus | Nil |  |  |  |  |  |  | -51 | -40 | -16 | 1 | 8.76 | | < 0.001 | 1.000 |
| Unlabeled | Nil |  |  |  |  |  |  | 30 | -22 | 2 | 1 | 8.52 | | < 0.001 | 1.000 |
| Left superior temporal gyrus | Nil |  |  |  |  |  |  | -39 | -25 | 5 | 3 | 7.32 | | < 0.001 | 1.000 |
| Right precentral gyrus | Nil |  |  |  |  |  |  | 39 | -22 | 59 | 5 | 6.69 | | 0.001 | 1.000 |
| Left cerebellum crus IV and V | Nil |  |  |  |  |  |  | -18 | -31 | -28 | 1 | 6.57 | | 0.001 | 1.000 |
| Left calcarine sulcus | Nil |  |  |  |  |  |  | -3 | -85 | -7 | 2 | 6.40 | | 0.001 | 1.000 |
| Right middle temporal gyrus | Nil |  |  |  |  |  |  | 63 | -46 | 11 | 1 | 6.38 | | 0.001 | 1.000 |
| Right cuneus | Nil |  |  |  |  |  |  | 12 | -82 | 41 | 1 | 6.18 | | 0.001 | 1.000 |
|  | | | | | | | | | | | | | | | |
| **Intact > Impaired** | | | | | | | | | | | | | | | |
| Right fusiform gyrus | 33 | -4 | -31 | 1 | 6.34 | < 0.001 | 1.000 | Nil |  |  |  |  | |  |  |
| Right medial frontal gyrus | 9 | 53 | 8 | 3 | 6.20 | < 0.001 | 1.000 | Nil |  |  |  |  | |  |  |
| Right anterior cingulum | Nil |  |  |  |  |  |  | 15 | 50 | 11 | 1 | 6.71 | | 0.001 | 1.000 |
| Unlabeled | Nil |  |  |  |  |  |  | 6 | -37 | -46 | 1 | 6.70 | | 0.001 | 1.000 |
| Right inferior frontal gyrus (triangular part) | Nil |  |  |  |  |  |  | 48 | 23 | -1 | 2 | 6.43 | | 0.001 | 1.000 |
| Right middle temporal gyrus | Nil |  |  |  |  |  |  | 60 | -13 | -19 | 1 | 6.30 | | 0.001 | 1.000 |
| Right middle cingulum | Nil |  |  |  |  |  |  | 9 | 29 | 32 | 1 | 5.95 | | 0.001 | 1.000 |
|  | | | | | | | | | | | | | | | |
| **Symptom awareness > Control stimuli** | | | | | | | | | | | | | | | |
| **Impaired > Intact** | | | | | | | | | | | | | | | |
| Left superior temporal gyrus | -54 | -31 | 17 | 1 | 8.45 | < 0.001 | 0.971 | -54 | -31 | 17 | 1 | 6.48 | | 0.001 | 1.000 |
| Right cerebellum crus IX | Nil |  |  |  |  |  |  | 18 | -52 | -43 | 1 | 5.91 | | 0.001 | 1.000 |
|  | | | | | | | | | | | | | | | |
| **Intact > Impaired** | | | | | | | | | | | | | | | |
| Left fusiform gyrus | -36 | -10 | -31 | 1 | 7.38 | < 0.001 | 0.987 | Nil |  |  |  |  | |  |  |
| Left superior orbital frontal gyrus | -18 | 50 | -4 | 1 | 6.28 | < 0.001 | 1.000 | Nil |  |  |  |  | |  |  |
| Right precentral gyrus | 63 | -4 | 32 | 1 | 5.87 | 0.001 | 1.000 | Nil |  |  |  |  | |  |  |
| Right middle frontal gyrus | 48 | 14 | 47 | 1 | 5.80 | 0.001 | 1.000 | Nil |  |  |  |  | |  |  |
| Right posterior cingulum | 3 | -37 | 14 | 1 | 5.70 | 0.001 | 1.000 | Nil |  |  |  |  | |  |  |
| Unlabeled | Nil |  |  |  |  |  |  | 18 | 2 | 35 | 3 | 13.09 | | < 0.001 | 0.963 |
| Unlabeled | Nil |  |  |  |  |  |  | 24 | -16 | 14 | 1 | 10.41 | | < 0.001 | 0.994 |
| Right precentral gyrus | Nil |  |  |  |  |  |  | 57 | -7 | 44 | 2 | 8.21 | | < 0.001 | 1.000 |
| Unlabeled | Nil |  |  |  |  |  |  | 30 | -31 | 14 | 1 | 6.76 | | 0.001 | 1.000 |
| Right postcentral gyrus | Nil |  |  |  |  |  |  | 60 | -4 | 32 | 1 | 5.96 | | 0.001 | 1.000 |
|  | | | | | | | | | | | | | | | |
| **Need for treatment > Control stimuli** | | | | | | | | | | | | | | | |
| **Impaired > Intact** | | | | | | | | | | | | | | | |
| Left cerebellar lobule VIIb | Nil |  |  |  |  |  |  | -12 | -70 | -43 | 1 | 7.00 | | < 0.001 | 1.000 |
| Unlabeled | Nil |  |  |  |  |  |  | 9 | -28 | 23 | 1 | 6.88 | | < 0.001 | 1.000 |
| Left putamen | Nil |  |  |  |  |  |  | -27 | -7 | 14 | 1 | 5.92 | | 0.001 | 1.000 |
|  | | | | | | | | | | | | | | | |
| **Intact > Impaired** | | | | | | | | | | | | | | | |
| Right medial orbital frontal gyrus | 12 | 47 | -4 | 4 | 8.59 | < 0.001 | 0.983 | Nil |  |  |  |  | |  |  |
| Left inferior temporal gyrus | -51 | -31 | -16 | 1 | 7.81 | < 0.001 | 0.995 | -51 | -31 | -16 | 1 | 7.93 | | < 0.001 | 1.000 |
| Left middle orbital frontal gyrus | -30 | 38 | -13 | 1 | 6.80 | < 0.001 | 0.999 | -30 | 38 | -13 | 1 | 7.06 | | < 0.001 | 1.000 |
| Left precuneus | -12 | -49 | 14 | 1 | 6.76 | < 0.001 | 1.000 | Nil |  |  |  |  | |  |  |
| Left medial orbital frontal gyrus | 0 | 32 | -13 | 1 | 6.76 | < 0.001 | 1.000 | 0 | 32 | -13 | 1 | 7.61 | | < 0.001 | 1.000 |
| Left anterior cingulum | -18 | 50 | -1 | 2 | 6.06 | < 0.001 | 1.000 | Nil |  |  |  |  | |  |  |
| Left medial orbital frontal gyrus | -9 | 41 | -7 | 1 | 5.70 | 0.001 | 1.000 | -9 | 41 | -7 | 2 | 6.22 | | 0.001 | 1.000 |
| Right medial orbitofrontal gyrus | Nil |  |  |  |  |  |  | 9 | 47 | -4 | 22 | 16.05 | | < 0.001 | 0.440 |
| Right putamen | Nil |  |  |  |  |  |  | 24 | -10 | 8 | 4 | 8.07 | | < 0.001 | 1.000 |
| Right thalamus | Nil |  |  |  |  |  |  | 24 | -19 | 8 | 1 | 7.93 | | < 0.001 | 1.000 |
| Right anterior cingulum | Nil |  |  |  |  |  |  | 9 | 23 | 26 | 2 | 7.26 | | < 0.001 | 1.000 |
| Left medial orbitofrontal gyrus | Nil |  |  |  |  |  |  | -12 | 32 | -10 | 1 | 6.79 | | 0.001 | 1.000 |
| Left superior orbitofrontal gyrus | Nil |  |  |  |  |  |  | -15 | 35 | -16 | 1 | 6.53 | | 0.001 | 1.000 |
| Right insula | Nil |  |  |  |  |  |  | 42 | 11 | -1 | 3 | 6.36 | | 0.001 | 1.000 |
| Unlabeled | Nil |  |  |  |  |  |  | -12 | 17 | 23 | 1 | 6.24 | | 0.001 | 1.000 |
| Left anterior cingulum | Nil |  |  |  |  |  |  | 0 | 35 | 23 | 1 | 6.01 | | 0.001 | 1.000 |
|  | | | | | | | | | | | | | | | |
| ***SUD subgroup only*** | | | | | | | | | | | | | | | |
| **Illness-related > Control stimuli** | | | | | | | | | | | | | | | |
| **Impaired > Intact** | | | | | | | | | | | | | | | |
| Unlabeled | -18 | -7 | 38 | 1 | 5.58 | 0.001 | 1.000 | Nil |  |  |  |  | |  |  |
| Unlabeled | -18 | -40 | 20 | 1 | 5.22 | 0.001 | 1.000 | Nil |  |  |  |  | |  |  |
| Left inferior temporal gyrus | Nil |  |  |  |  |  |  | -36 | -1 | -37 | 1 | 6.75 | | 0.001 | 1.000 |
| Left precuneus | Nil |  |  |  |  |  |  | -15 | -49 | 11 | 1 | 6.56 | | 0.001 | 1.000 |
| Left thalamus | Nil |  |  |  |  |  |  | -24 | -24 | 14 | 1 | 5.95 | | 0.001 | 1.000 |
|  | | | | | | | | | | | | | | | |
| **Intact > Impaired** | | | | | | | | | | | | | | | |
| Left superior frontal gyrus | Nil |  |  |  |  |  |  | -21 | -7 | 50 | 2 | 7.13 | | < 0.001 | 1.000 |
| Left inferior frontal gyrus (triangular part) | Nil |  |  |  |  |  |  | -42 | 35 | 26 | 1 | 6.25 | | 0.001 | 1.000 |
|  | | | | | | | | | | | | | | | |
|  | | | | | | | | | | | | | | | |
| **General illness awareness > Control stimuli** | | | | | | | | | | | | | | | |
| **Impaired > Intact** | | | | | | | | | | | | | | | |
| Right superior frontal gyrus | 18 | 56 | 8 | 2 | 8.92 | < 0.001 | 0.988 | Nil |  |  |  |  | |  |  |
| Left inferior frontal gyrus (triangular part) | Nil |  |  |  |  |  |  | 24 | -4 | 32 | 2 | 7.98 | | < 0.001 | 1.000 |
| Right caudate | Nil |  |  |  |  |  |  | 21 | 2 | 29 | 1 | 7.14 | | < 0.001 | 1.000 |
| Unlabeled | Nil |  |  |  |  |  |  | 21 | -22 | 38 | 1 | 6.70 | | 0.001 | 1.000 |
| Left anterior cingulum | Nil |  |  |  |  |  |  | -3 | 32 | -4 | 4 | 6.61 | | 0.001 | 1.000 |
| Left caudate | Nil |  |  |  |  |  |  | -18 | 23 | 20 | 1 | 6.34 | | 0.001 | 1.000 |
| Left insula | Nil |  |  |  |  |  |  | -39 | 2 | 11 | 1 | 6.15 | | 0.001 | 1.000 |
| Unlabeled | Nil |  |  |  |  |  |  | 12 | 14 | 23 | 1 | 6.04 | | 0.001 | 1.000 |
|  | | | | | | | | | | | | | | | |
| **Intact > Impaired** | | | | | | | | | | | | | | | |
| Right superior temporal gyrus | 63 | -1 | -7 | 1 | 5.39 | 0.001 | 1.000 | Nil |  |  |  |  | |  |  |
| Unlabeled | Nil |  |  |  |  |  |  | -21 | -13 | 50 | 1 | 6.20 | | 0.001 | 1.000 |
| Left inferior frontal gyrus (triangular part) | Nil |  |  |  |  |  |  | -42 | 35 | 26 | 2 | 6.13 | | 0.001 | 1.000 |
| Unlabeled | Nil |  |  |  |  |  |  | 18 | -22 | 53 | 1 | 6.02 | | 0.001 | 1.000 |
|  | | | | | | | | | | | | | | | |
| **Symptom awareness > Control stimuli** | | | | | | | | | | | | | | | |
| **Impaired > Intact** | | | | | | | | | | | | | | | |
| Unlabeled | -9 | -28 | 23 | 1 | 6.46 | < 0.001 | 1.000 | Nil |  |  |  |  | |  |  |
| Left calcarine sulcus | -27 | -61 | 14 | 2 | 6.15 | < 0.001 | 1.000 | Nil |  |  |  |  | |  |  |
| Left putamen | -24 | -13 | 14 | 1 | 5.25 | 0.001 | 1.000 | Nil |  |  |  |  | |  |  |
| Unlabeled | Nil |  |  |  |  |  |  | -18 | -10 | 41 | 1 | 7.75 | | < 0.001 | 1.000 |
| Unlabeled | Nil |  |  |  |  |  |  | -30 | -13 | 29 | 1 | 6.61 | | 0.001 | 1.000 |
| Right inferior temporal gyrus | Nil |  |  |  |  |  |  | 42 | -52 | -10 | 1 | 5.90 | | 0.001 | 1.000 |
|  | | | | | | | | | | | | | | | |
| **Intact > Impaired** | | | | | | | | | | | | | | | |
| Left superior frontal gyrus | Nil |  |  |  |  |  |  | -21 | -7 | 50 | 1 | 7.96 | | < 0.001 | 1.000 |
| Left insula | Nil |  |  |  |  |  |  | -39 | -7 | 5 | 1 | 6.38 | | 0.001 | 1.000 |
| Right insula | Nil |  |  |  |  |  |  | 45 | 11 | -1 | 1 | 6.28 | | 0.001 | 1.000 |
| Right middle cingulum | Nil |  |  |  |  |  |  | 9 | -25 | 41 | 1 | 6.19 | | 0.001 | 1.000 |
| Unlabeled | Nil |  |  |  |  |  |  | 18 | -13 | 50 | 1 | 6.17 | | 0.001 | 1.000 |
|  | | | | | | | | | | | | | | | |
| **Need for treatment > Control stimuli** | | | | | | | | | | | | | | | |
| **Impaired > Intact** | | | | | | | | | | | | | | | |
| Left middle occipital gyrus | -33 | -73 | 5 | 1 | 6.87 | < 0.001 | 0.999 | Nil |  |  |  |  | |  |  |
| Left operculum of the rolandic gyrus | -36 | -31 | 20 | 1 | 6.87 | < 0.001 | 0.999 | Nil |  |  |  |  | |  |  |
| Left middle occipital gyrus | -36 | -76 | -1 | 1 | 6.59 | < 0.001 | 1.000 | Nil |  |  |  |  | |  |  |
| Left calcarine sulcus | -24 | -58 | 14 | 1 | 6.50 | < 0.001 | 1.000 | -24 | -58 | 14 | 1 | 6.22 | | 0.001 | 1.000 |
| Unlabeled | -21 | -34 | 23 | 2 | 5.93 | 0.001 | 1.000 | Nil |  |  |  |  | |  |  |
| Right hippocampus | 36 | -25 | -13 | 1 | 5.85 | 0.001 | 1.000 | Nil |  |  |  |  | |  |  |
| Left calcarine sulcus | -15 | -49 | 8 | 1 | 5.83 | 0.001 | 1.000 | Nil |  |  |  |  | |  |  |
| Left fusiform gyrus | -33 | -73 | -10 | 1 | 5.49 | 0.001 | 1.000 | Nil |  |  |  |  | |  |  |
| Right middle frontal gyrus | 39 | 11 | 56 | 1 | 5.21 | 0.001 | 1.000 | Nil |  |  |  |  | |  |  |
|  | | | | | | | | | | | | | | | |
| **Intact > Impaired** | | | | | | | | | | | | | | | |
| Unlabeled | -30 | -52 | 26 | 1 | 5.32 | 0.001 | 1.000 | Nil |  |  |  |  | |  |  |
| Right precuneus | Nil |  |  |  |  |  |  | 6 | -52 | 20 | 9 | 13.08 | | < 0.001 | 0.988 |
| Right thalamus | Nil |  |  |  |  |  |  | 18 | -10 | 8 | 1 | 6.76 | | 0.001 | 1.000 |
| Right precuneus | Nil |  |  |  |  |  |  | 9 | -52 | 71 | 2 | 6.49 | | 0.001 | 1.000 |
| Unlabeled | Nil |  |  |  |  |  |  | -45 | -25 | 26 | 3 | 6.28 | | 0.001 | 1.000 |
| Left superior frontal gyrus | Nil |  |  |  |  |  |  | -21 | -7 | 50 | 3 | 6.14 | | 0.001 | 1.000 |
| Right supramarginal gyrus | Nil |  |  |  |  |  |  | 57 | -28 | 29 | 1 | 6.13 | | 0.001 | 1.000 |
| Note: Threshold: *p* < 0.001, uncorr., 0 voxels;  No suprathreshold peak in association with intact > impaired was identified  **p* < 0.05 | | | | | | | | | | | | | | | |
